# Whole Genome Sequencing for Genomics-Guided Investigations of *Escherichia coli* O157:H7 Outbreaks

**DOI:** 10.3389/fmicb.2016.00985

**Published:** 2016-06-30

**Authors:** Brigida Rusconi, Fatemeh Sanjar, Sara S. K. Koenig, Mark K. Mammel, Phillip I. Tarr, Mark Eppinger

**Affiliations:** ^1^South Texas Center for Emerging Infectious Diseases, University of Texas at San AntonioSan Antonio, TX, USA; ^2^Department of Biology, University of Texas at San AntonioSan Antonio, TX, USA; ^3^Center for Food Safety and Applied Nutrition, U.S. Food and Drug AdministrationLaurel, MD, USA; ^4^Department of Pediatrics, Washington University School of MedicineSt. Louis, MO, USA

**Keywords:** *Escherichia coli*, O157:H7, EHEC, phylogenomics, outbreaks, single nucleotide polymorphism, genomic epidemiology, whole genome sequence typing

## Abstract

Multi isolate whole genome sequencing (WGS) and typing for outbreak investigations has become a reality in the post-genomics era. We applied this technology to strains from *Escherichia coli* O157:H7 outbreaks. These include isolates from seven North America outbreaks, as well as multiple isolates from the same patient and from different infected individuals in the same household. Customized high-resolution bioinformatics sequence typing strategies were developed to assess the core genome and mobilome plasticity. Sequence typing was performed using an in-house single nucleotide polymorphism (SNP) discovery and validation pipeline. Discriminatory power becomes of particular importance for the investigation of isolates from outbreaks in which macrogenomic techniques such as pulse-field gel electrophoresis or multiple locus variable number tandem repeat analysis do not differentiate closely related organisms. We also characterized differences in the phage inventory, allowing us to identify plasticity among outbreak strains that is not detectable at the core genome level. Our comprehensive analysis of the mobilome identified multiple plasmids that have not previously been associated with this lineage. Applied phylogenomics approaches provide strong molecular evidence for exceptionally little heterogeneity of strains within outbreaks and demonstrate the value of intra-cluster comparisons, rather than basing the analysis on archetypal reference strains. Next generation sequencing and whole genome typing strategies provide the technological foundation for genomic epidemiology outbreak investigation utilizing its significantly higher sample throughput, cost efficiency, and phylogenetic relatedness accuracy. These phylogenomics approaches have major public health relevance in translating information from the sequence-based survey to support timely and informed countermeasures. Polymorphisms identified in this work offer robust phylogenetic signals that index both short- and long-term evolution and can complement currently employed typing schemes for outbreak ex- and inclusion, diagnostics, surveillance, and forensic studies.

## Introduction

Microbial pathogens with a foodborne etiology present major challenges to public health. *Escherichia coli* has been divided into different pathovars based on key virulence factors that define their pathogenicity (Sadiq et al., 2014). One particularly daunting pathovar among the Shiga toxin producing *E. coli* (STEC) are strains of the enterohemorrhagic O157:H7 serotype, which can be transmitted by a variety of vehicles, and causes serious human disease (Tarr et al., 2005). Currently, there is no effective treatment or prophylaxis for hemolytic uremic syndrome (HUS) (Goldwater and Bettelheim, 2012), and use of antibiotics is not indicated (Freedman et al., 2016). Since its discovery in 1982, this lineage has rapidly evolved from a rare serotype into the now globally dominant enterohemorrhagic *E. coli* (EHEC) serotype. A remarkable feature is its low infectious dose; it is estimated that 10–100 colony-forming units (CFUs) are sufficient to cause disease (Tilden et al., 1996; Tuttle et al., 1999) For the above reasons, prevention of human infection is critical, and early identification of outbreaks is highly worthwhile. However, only rudimentary information exists regarding the genomic heterogeneity that can be expected within outbreaks (STEC Outbreaks). Moreover, current typing schemes, such as pulse field gel electrophoresis (PFGE) and multiple locus variable number of tandem repeats analysis (MLVA), often lack the resolution to differentiate organisms that form tightly clonal phylogenetic clusters within the O157:H7 clade (Eppinger et al., 2011b; Turabelidze et al., 2013; Underwood et al., 2013; Rusconi and Eppinger, 2016). Additionally, PFGE is subject to technological and interpretation challenges (Davis et al., 2003).

Increasing technologic economies offer new opportunities for sequence-based typing of microbial pathogens for public health purposes (den Bakker et al., 2014; Joensen et al., 2014; Leekitcharoenphon et al., 2014; Holmes et al., 2015). While it would be ideal to refer a clinical strain's sequence to a reference, of the 445 publicly available genomes of *E. coli* O157:H7 and its close relative O55:H7 (O157:H7 Genomes) (Kulasekara et al., 2009; Zhou et al., 2010; Eppinger et al., 2011a, 2013; Sanjar et al., 2014, 2015), to date only 11 have been closed (Hayashi et al., 2001; Perna et al., 2001; Kulasekara et al., 2009; Zhou et al., 2010; Eppinger et al., 2011b, 2013; Kyle et al., 2012; Xiong et al., 2012; Latif et al., 2014; Sanjar et al., 2014, 2015; Cote et al., 2015). Whole genome sequencing (WGS) can provide the necessary resolution power to investigate apparent single source outbreaks (Eppinger et al., 2011b; Hasan et al., 2012; Turabelidze et al., 2013) because the granularity of WGS data provides considerable confidence in assigning like vs. not-like status to two potentially linked pathogens (Gilchrist et al., 2015). Such data can also link pathogens to vehicles or environmental isolates most precisely (Bentley and Parkhill, 2015). WGS can offer additional advantages: serotypes and virulence loci within pathogens can be identified (Scheutz et al., 2012; Leekitcharoenphon et al., 2014; Lambert et al., 2015; Klemm and Dougan, 2016), and case management might theoretically be risk-optimized.

Optimization of *E. coli* O157:H7 sequence analysis methodologies depend on the scientific and epidemiologic inquiries and the data being analyzed. Pettengill et al. evaluated a number of single nucleotide polymorphism (SNP) predicting tools and phylogenetic methodologies in prokaryotes and concluded that a reference-based approach, which accommodates missing data as well as infers phylogenetic reconstruction, is the most appropriate (Pettengill et al., 2014). Such a reference-based approach was recently used by the Alberta Provincial Laboratory for Public Health to study *E. coli* O157:H7 outbreaks together with virulence profiling and other molecular methods (Berenger et al., 2015). No specific virulence pattern distinguished the outbreak strains from sporadic strains (Berenger et al., 2015). Recent studies have expanded WGS typing to globally distributed strains and identified geographical genomic structuring based on distribution of *stx*-converting phage integration sites and SNPs (Mellor et al., 2015; Strachan et al., 2015) and provided a more detailed subtyping of *E. coli* O157:H7 (Griffing et al., 2015). However, clarity can also be gained by comparing closely related isolates to each other, rather than to reference strains (Leopold et al., 2009; Turabelidze et al., 2013).

Here we adapt WGS to a specifically developed SNP-based pipeline for the high resolution typing of *E. coli* O157:H7 by identifying SNPs within the core genome. In addition to SNP analysis in the core genome we assessed plasticity in the mobilome by LS-BSR and plasmid comparison (phages and plasmids) (Eppinger et al., 2011a,b, 2014; Hasan et al., 2012; Jenkins et al., 2015). We tested this pipeline on isolates from seven retrospectively analyzed EHEC O157:H7 outbreaks, six intra-household cases, and five clinical “plate-mate” pairs, i.e., colonies from the same primary isolation plate from the clinical laboratory.

## Materials and methods

### Strains in study

We compared human isolates (Supplemental Table 1) of nine phylogenetic clades (Manning et al., 2008), so as to place the strains in the overall *E. coli* O157:H7 phylogenetic context. Strain-associated metadata of analyzed *E. coli* O157:H7 are provided in Supplemental Table 1. Outbreak strains were defined as a set of isolates from different cases of infection arising from a single point source, as determined by local health jurisdictions and/or the Centers for Disease Control and Prevention. Intra-household cluster strains were recovered from siblings within a household whose infections were not linked to a recognized outbreak. Because intra-household clusters could reflect co-primary infections rather than secondary transmission, we selected such pairings from among our strain set collection (Cornick et al., 2002; Besser et al., 2007) on the basis of prolonged intervals (4–6 days) between cases, so as to increase the likelihood that genomic diversity might emerge secondary to inter-host transmission. Plate mates are pairs of isolates from the same sorbitol-MacConkey agar plate used in clinical laboratories to diagnose the infection.

### Bioinformatic analyses for polymorphisms discovery in core genome and mobilome

**Figure 1:**
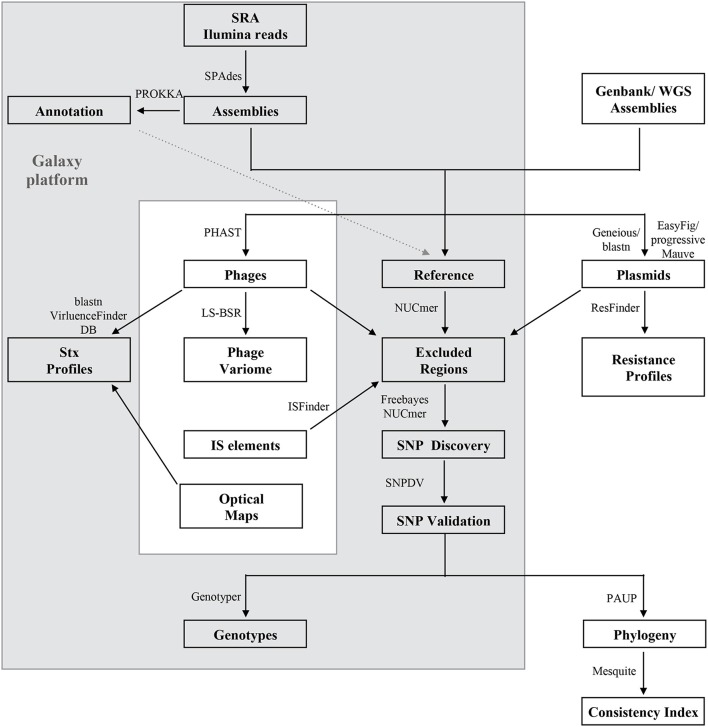
**Bioinformatics analyses of the ***E. coli*** O157:H7 core and mobile genome**. The majority of the analyses were performed on Galaxy (Goecks et al., 2010), an open-source web-based bioinformatics platform (gray box). Illumina reads, draft or closed genomes were retrieved from the NCBI SRA or WGS sequence repositories (Supplemental Table 1). Reads were assembled with SPAdes v 3.5.0 (Bankevich et al., 2012), and annotated with PROKKA v 1.11.0 (Seemann, 2014). A reference genome was selected for each outbreak analysis and distinct regions were excluded from SNP validation as follows: Repeats with NUCmer (Delcher et al., 2003), phages with PHAST (Zhou et al., 2011) plasmids by querying against a custom *E. coli* plasmid database and IS elements from ISFinder (Siguier et al., 2006). Optical maps were generated to facilitate accurate prophage and mobilome profiling (Eppinger et al., 2011b). Read based SNP discovery was based on Bowtie2 alignment (Langmead and Salzberg, 2012) and subsequent Freebayes SNP calling (Garrison and Marth, 2012) and for closed or draft genomes on NUCmer (Delcher et al., 2003), followed by blast- and PCR-based SNPs validations to curate for false-positives with strategies detailed in (Eppinger et al., 2011b, 2014). The identified SNPs were genotyped and used for phylogenetic reconstruction by maximum parsimony of the outbreaks with PAUP v4.0a146 (Wilgenbusch and Swofford, 2003). Consistency index and majority consensus trees were built with Mesquite (Maddison and Maddison, 2015). The mobilome was analyzed in regards to phage content with large scale-blast score ratio (LS-BSR) (Sahl et al., 2014) and computed matrices visualized with MeV (Saeed et al., 2003). Shiga toxins were identified by discontiguous megablast (Buhler, 2001; Ma et al., 2002) against the VirulenceFinder (Joensen et al., 2014) database. Plasmids were identified after alignment of draft genomes to the closest closed reference plasmid in Geneious (vR9) (Kearse et al., 2012). Unmapped remaining contigs were queried against NCBI nr plasmids by discontiguous megablast (Buhler, 2001; Ma et al., 2002). Identified plasmid homologs were compared and visualized with EasyFig (Sullivan et al., 2011) by tblastx (Altschul et al., 1990) or progressiveMauve (Darling et al., 2010). Plasmids and chromosomes were further surveyed for presence of resistance loci using ResFinder (Kleinheinz et al., 2014).

Developed bioinformatics workflows, methods and principles for SNP discovery and core and accessory genome analyses performed in this study are described in Figure 1 with external tools referenced in the legend. Multinucleotide insertions and deletions of polymorphic bases were not considered SNPs. To classify SNPs we mapped the annotation from the *de novo* annotated references with PROKKA and Prodigal ORF prediction (Hyatt et al., 2010), or the deposited annotation for EC4115 (Eppinger et al., 2011b). The core genome was defined as the set of genic and intragenic regions that were not repeated, did not contain phages, IS elements, or plasmid regions. Briefly for SNP discovery, reads were aligned with Bowtie2 (Langmead and Salzberg, 2012) to designated reference genomes. Resulting alignments were processed with Freebayes (Garrison and Marth, 2012) with the following threshold settings: mapping quality 30, base quality 20, coverage 30, and allelic frequency 0.9. To account for false positive calls we used several SNP curation strategies: (i) Reference reads were mapped against the reference genome and false positives were identified by Freebayes with the settings described above; (ii) If reads were not available, the post-assembly workflow created a reference-based NUCmer alignment and extracted SNPs with delta-filter and show-snps distributed with the MUMmer package (Delcher et al., 2003). SNP occurring in the excluded regions were removed. Cataloged SNPs from each genome were merged into a single SNP panel, and allelic status and chromosomal position were recorded. Curated SNPs were further processed by extracting the surrounding nucleotides (40 nt) and blastn against the query genomes (Altschul et al., 1990). Resulting alignments were parsed to remove SNP locations derived from ambiguous hits (≥2), non-uniformly distributed regions, and insertion or deletion events, as previously described (Myers et al., 2009; Morelli et al., 2010; Eppinger et al., 2011b, 2014; Vogler et al., 2011; Hasan et al., 2012).

### Optical maps

Optical mapping facilitated accurate phage profiling (Kotewicz et al., 2008). In total 12 maps were generated (Supplemental Table 1), either prepared by OpGen or contributed by FDA (Eppinger et al., 2011b, 2014). After gentle lysis and dilution, the extracted genomic DNA molecules from each strain were spread and immobilized onto derivatized glass slides. The genomic DNA was then digested with BamH1 restriction enzyme maintaining the DNA fragment order. Using the Argus™ Instrument, the DNA fragments were stained with YOYO-1 fluorescent dye and photographed using a fluorescent microscope interfaced with a digital camera. The optical data was converted to digital data, which defines single molecule restriction maps. Physical maps were complemented with *in silico* maps of other outbreak strains, and comparatively analyzed in MapSolver™ Optical Map Analysis software (Latreille et al., 2007; Zhou et al., 2007).

### SNP PCR validation

SNPs in four isolates from two outbreaks for which we possessed archived cultures were subjected to PCR confirmation using primer pair (89750-F 5′- ACA ACG ATA TGA TCG ACC AGC, 89750-R 5′- TTG TAC AGA AGA CCA TGC TCG) and (27005-F 5′- AGA GTA CGG ATT CAC CTT GCC, 27005-R 5′- AGT CAG GCA ATT CCT CGT GG, 78298-F 5′- AGT CAT TAC CAG GAA CAG CAG 78298-R 5′- TGT TCG AGA TTC TGG TGA GTG) for strains from the Battle Ground Lake and Finley School District outbreak, respectively. Resulting amplicons were Sanger sequenced.

### Multi drug resistance (MDR) profiling

Susceptibility to amikacin, ampicillin, amoxicillin-clavulanic acid, cefoxitin, ceftiofur, ceftriaxone, chloramphenicol, ciprofloxacin, gentamicin, kanamycin, nalidixic acid, streptomycin, sulfisoxazole, tetracycline, and trimethoprim-sulfamethoxazole was assessed at FDA according to the NARMS methodology and manufacturer's instructions with the Sensititre automated system (Trek Diagnostic Systems, Westlake, OH) (Zhao et al., 2008). Resistance was determined by comparing MICs to Clinical and Laboratory Standards Institute (CLSI) values (Institute, 2013).

## Results and discussion

### Epidemiology of investigated strains

We analyzed 36 strains from seven US outbreaks as recognized by the CDC that occurred between 1998 and 2009 (Supplemental Table 1): (1) 11 children were infected after consumption of contaminated ground beef tacos in the Finley School District (FS) in 1998; (2) 28 swimmers at Battle Ground (BL) Lake State Park, WA, and eight secondary cases were infected in 1999; (3) 81 cases were attributed to lettuce served at multiple outlets of a taco chain (Taco John) in 2006; (4) 71 people were infected in a multistate outbreak after eating at Taco Bell (TB) in 2006; though the vehicle was not identified (Taco Bell); (5) 21 infections were attributed to a prolonged multi-state outbreak linked to the consumption of Totino's or Jeno's contaminated pepperoni pizza (Totino's pizza) (TP) in 2007; (6) 76 cases were attributed to a nationwide outbreak of contaminated cookie dough (CD) (Cookie dough) in 2009; (7) 26 patients from eight states were infected by beef traced to Fairbank Farms (FF) in 2009 (Fairbank Farms).

**Figure 2:**
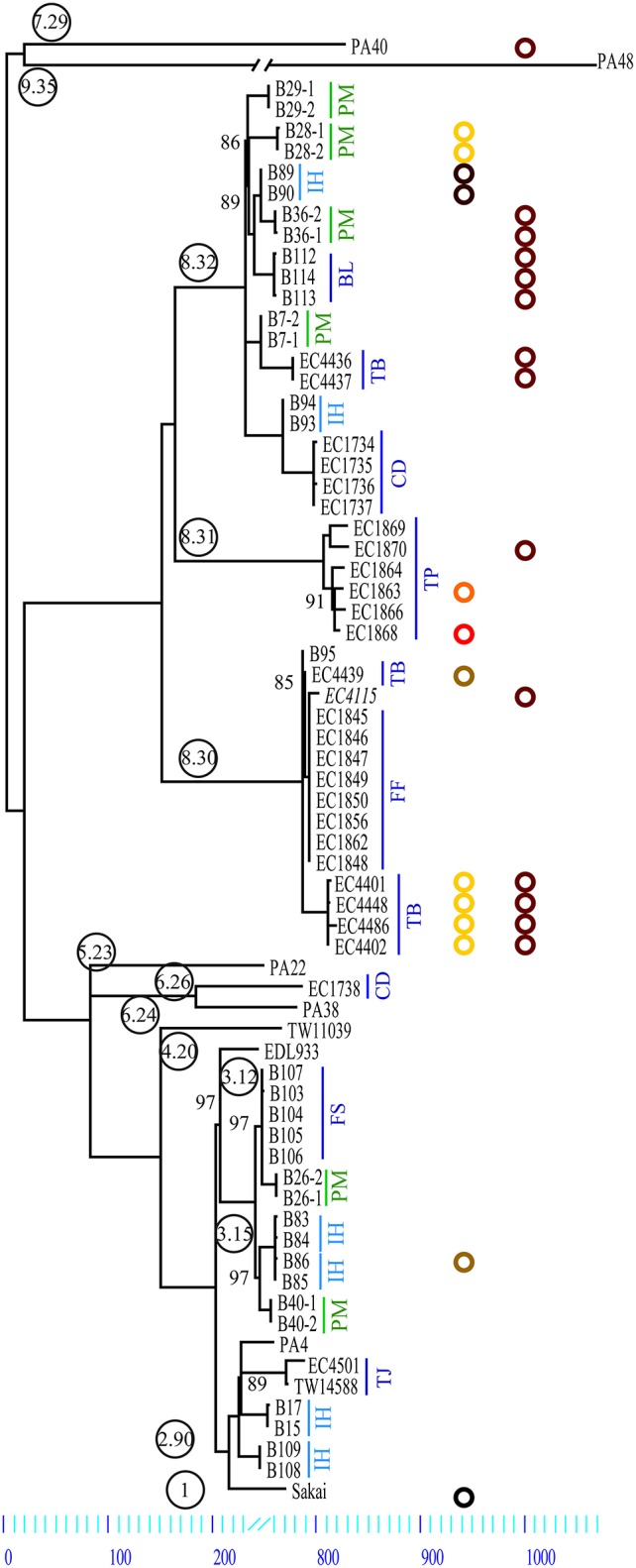
**Maximum parsimony tree of ***E. coli*** O157:H7**. Comparison of 70 genomes yielded a total of 3313 SNPs of which 1266 were parsimony informative. The tree shown is a majority-consensus tree of 28 equally parsimonious trees with a consistency index of 0.998. Trees were recovered using a heuristic search in Paup 4.0b10 (Wilgenbusch and Swofford, 2003). Only nodes with bootstrap values below 100 are listed. Phylogenetic clade association is provided in circled numbers (Manning et al., 2006). Strains investigated are comprised of PM, Plate mates; IH, intrahousehold infections; BL, Battle Ground Lake; CD, Cookie Dough; FS, Finley School District; FF, Fairbank Farms; TB, Taco Bell; TJ, Taco John; and TP, Totino's Pizza. Our plasmid survey confirmed that all strains carry the lineage-specific plasmid pO157. Further we identified other plasmids with a size range from 34 to 78 kb. Plasmid type prevalence is represented in colored circles: p78 (yellow), p63 (orange), p55 (light brown), p39 (red), p36 (brown), p34 (dark brown), and a small 3.3 kb plasmid (black) (Makino et al., 1998). Plasmid p36 is homologous to pEC4115 (Eppinger et al., 2011b).

We further studied (#12) strains from six intrahousehold illnesses (IH), in which the pathogen probably spread between patients based on the long intervals between onset in the individual family members (Supplemental Table 1). Though we cannot exclude the possibility of infection from the same source (co-primary). The median incubation period of *E. coli* O157:H7 infections is 3 days (Bell et al., 1994), and onsets ranged between 4 and 6 days. We also studied pairs of isolates from the same primary plate in the clinical laboratory (plate-mates, PM) from six patients (Figure 2). The clinical strains were compared to strains representing the nine phylogenetic clades reported by Manning et al. (2008) (Figure 2, Supplemental Table 1).

### Core genome phylogeny

We applied WGS typing strategies to determine the phylogenetic relatedness of the individual outbreak strains in the context of outbreak etiology, and to place them into the larger phylogenomic framework of the *E. coli* O157:H7 lineage (Leopold et al., 2009; Eppinger et al., 2011b; Dallman et al., 2015; Holmes et al., 2015; Jenkins et al., 2015). Of the 3313 SNPs identified in these 70 genomes, 2797 were intragenic and 516 were intergenic (Table 1, Supplemental Dataset 1). We observed significantly more SNPs in intergenic regions (chi square test, *p* < 10^−14^) than would be expected when considering the average intergenic frequency in EC4115 of 11.1% when compared to the percentage (15.5%) delineated from the cataloged SNPs. We note that, even though we excluded repeated regions and phages/mobilome during SNP discovery, thereby reducing the genome content by 20%, the coding to non-coding ratio of the remaining core genome remained stable. Homoplasy was negligible: only seven homoplastic SNPs were found dispersed throughout the chromosome (Supplemental Table 2) evidenced by a consistency index of 0.998. Of the seven homoplastic SNPs four are in *rpoS*, which is known to be highly polymorphic in *E. coli* O157:H7 (Uhlich et al., 2013). In line with our previous findings (Leopold et al., 2009; Eppinger et al., 2011a,b), SNPs were evenly distributed throughout the chromosome (Figure 2) without any mutational hot spots as found in other enteric pathogens (Hasan et al., 2012; Eppinger et al., 2014). From the cataloged SNP panel we delineated a total of 77 individual SNP genotypes. These genotypes represent only two-thirds of the 115 nodes in the tree (Figure 2, Supplemental Table 2), which can be attributed to the lack of terminal strain-specific SNPs (Figure 2). Among the cataloged 3313 SNPs, approximately one-third (#1266) is parsimony-informative. The SNPs in PA40 and PA48 are not strain-specific, but indicate the relative phylogenetic distance that separates these clade 7 and 9 strains from the other clades (Manning et al., 2008). As evidenced in the tree topology, approximately half of the parsimony non-informative SNPs (#1046) is introduced by reference strain PA48 from clade 9 (Figure 2, Supplemental Table 2). Among investigated strains PA48 is phylogenetically closest to the progenitor O55:H7 serotype (Feng et al., 1998; Manning et al., 2008; Zhou et al., 2010). This clade is also within the most ancient cluster of *E. coli* O157:H7 (Leopold et al., 2009) and higher SNP counts are indicative of more time to accrue mutations than in other phylogenetic groups that have emerged more recently.

**Table 1:** **SNPs characteristics for 70 ***E. coli*** O157:H7**.

	**Total**	**NSYN**	**NSYN/ NSYN**	**SYN**	**SYN/SYN**	**Intergenic**	**Genic**
SNPs	3313	1712	1	1083	1	516	2797
NI	2048	1054	1	656	1	335	1712
PI	1266	658	0	427	0	181	1085
Genes	1821	1264	1	891	1	0	2157
Stop gain	50	57	0	0	0	0	57
Stop loss	16	17	0	0	0	0	17
Hypothetical proteins	245	269	1	103	0	0	373
Transition	2254	1037	1	852	1	363	1891
Transversion	1064	676	1	231	1	155	909
Multiallelic	5	1	1	2	1	2	5

### Genomic epidemiology of north american outbreaks

**Figure 3:**
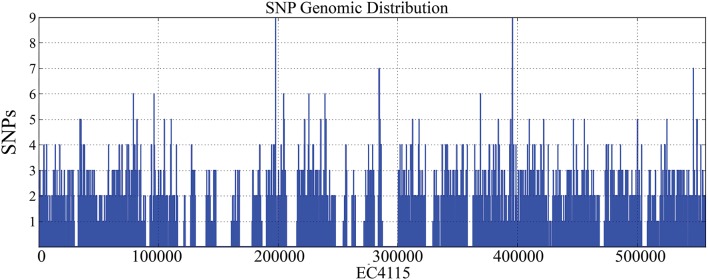
**Genome-wide distribution of SNPs**. Chromosomal positions of the 3313 identified SNPs were plotted along the EC4115 chromosome using a 1000 bp sliding window. We found SNPs dispersed throughout the chromosome providing no indication for mutational hotspots. Deserted regions lacking SNP calls correspond to excluded mobile regions, such as *stx*-prophages.

**Figure 4:**
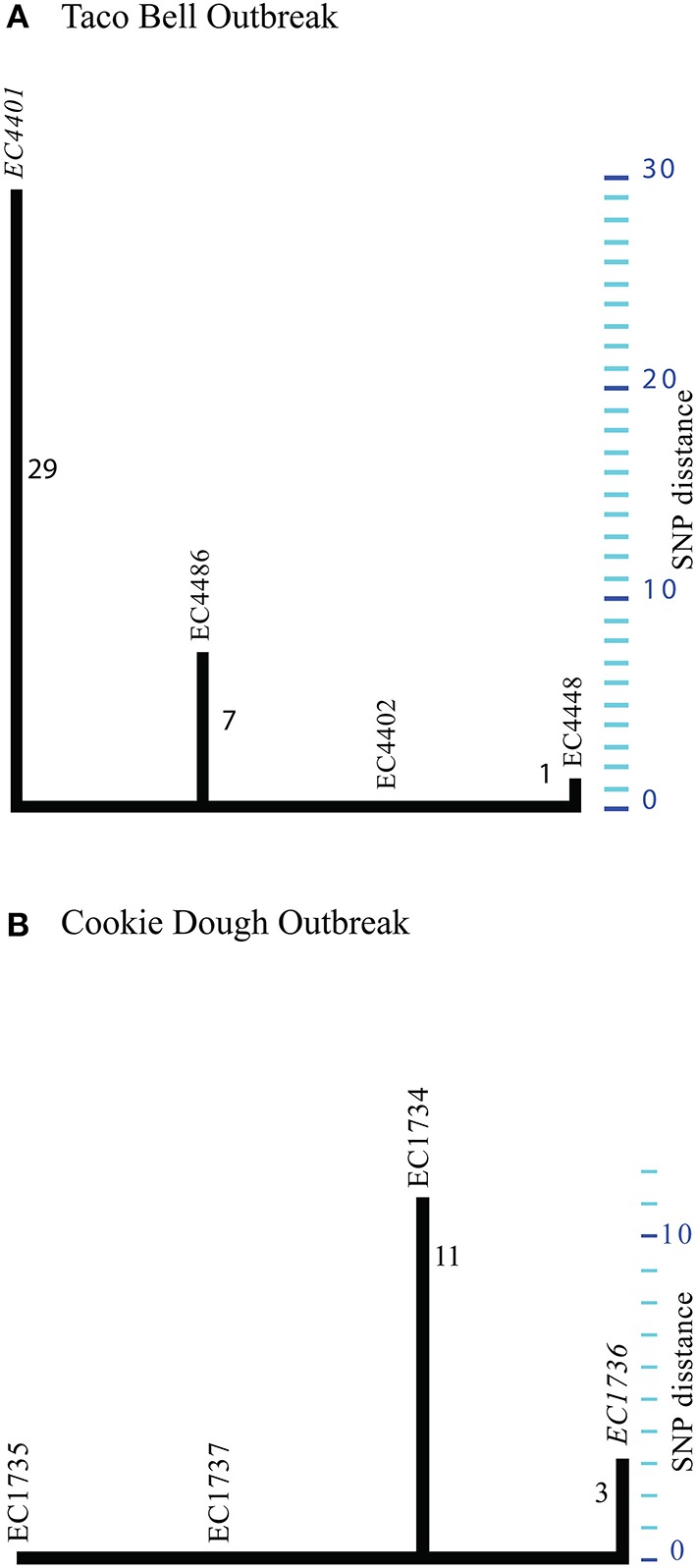
**Phylogenetic tree for Taco Bell (A) and Cookie dough (B) outbreak strains**. Maximum parsimony phylogeny computed from 37 TB and 14 CD SNPs identified in the respective outbreak associated strains. **(A)** By using a TB outbreak-specific reference (EC4401) we increased the phylogenetic resolution for these highly clonal strains. **(B)** Maximum parsimony phylogeny delineated from 14 SNPs discovered in the four CD outbreak associated strains. Strain EC1736 served as reference for the read based discovery. Majority of SNPs (#11) were specific to EC1734 and probably false positives from through assembly based discovery.

Guided by the established phylogenomic framework (Figure 2), we analyzed outbreak specific “genome” characteristics and polymorphic heterogeneity in seven different North American outbreaks using a common (EC4115), as well as outbreak-specific references. We specifically applied this dual reference genome approach to improve resolution power by enabling polymorphism discovery in parts of the core genome integral to the outbreak-associated strains, but not necessarily present in a more phylogenetic distant reference like EC4115 (Figure 2). We note here that our investigation of the 2006 Spinach (SP) outbreak revealed a number of subtle polymorphisms distinguishing all the recovered Maine isolates from the remainder of SP strains. Such subtle polymorphisms would have clearly evaded detection by using a reference from outside the SP outbreak (Eppinger et al., 2011b). In general we observed limited plasticity among related outbreak strains when compared to the closed reference genome EC4115 (Tables 2–**5**) (Eppinger et al., 2011b). Strains with increased SNP numbers were either from cases that were epidemiologically predicted to be outliers, or that could not be read-corrected. For example, the TB outbreak associated strains included four strains classified by CDC as temporal outliers (Supplemental Table 1), two (EC4436, EC4437) separated by 285 SNPs and one (EC4439) by 27 SNPs from the core outbreak cluster (Figure 2, Tables 2, 3). According to our SNP analysis, the remaining strain EC4448 should be considered to be derived from a single point source, even if this isolate is separated by a single homoplastic nonsynonymous (nsyn) SNP (Figures 2, 3, Tables 2, 3, Supplemental Table 2). Notably, *rpoS* carries this homoplastic stop codon mutation, which is known to be highly polymorphic in *E. coli* O157:H7; particularly in regards to premature stop codons that affect curli expression and biofilm formation (Uhlich et al., 2013). The same SNP was identified with an outbreak specific reference EC4401 in addition to multiple (#31) reference specific alleles (Figure 4A, Tables 2, 3, Supplemental Dataset 1). These SNPs were mainly located in intergenic (#30) regions and probably caused by over-predictions because of a lack of reads in the genome repository, and consequently inability to perform quality control. We observed the same phenomenon of over-prediction for the TJ outbreak strains, separated by 24 SNPs (Tables 2, 3, Supplemental Dataset 1); again no read data were available to us. We found the majority of predicted SNPs clustered mainly in close proximity either in intergenic regions or within the boundaries of the same gene, indicative of low quality sequence regions. Intragenic SNPs were identical to those found in EC4115, except for two additional SNPs in the *lac* repressor and in *rpoS* (Supplemental Dataset 1). The CD outbreak set underwent both contig and read-based discovery, which again over-predicted SNPs for EC1734 (no reads) due to a lack of reads for quality control (Figures 2, 4B, Tables 2, 3). Moreover, the production lot isolate EC1738 was placed on a distant branch (clade 6.26), separated from all human isolates tightly clustered in clade 8.30 (Figure 1, Table 2, Supplemental Table 1). Hence, we consider this strain as an outlier, which is phylogenetically unrelated to the case isolates. Among the outbreak-specific SNPs we detected one synonymous (syn) and two nsyn SNPs in EC1736, but the syn was also detected using EC4115 as a reference (Table 2, Supplemental Dataset 1). Archived strains were not available for this outbreak and we could therefore not confirm if EC1736 truly carries these 3 SNPs, which would question its inclusion into the outbreak.

**Table 2:** **Comparison of common vs. outbreak-specific reference genic SNPs**.

**Isolate**	**Sequences analyzed^a^**	**Synonymous SNPs in isolates, among backbone ORFs**			**Nonsynonymous SNPs in isolates, among backbone ORFs**	**Comments**
		**Compared to reference EC4115**	**Compared to outbreak strain**	**Compared to reference EC4115**	**Compared to outbreak strain**	
**FINLEY SCHOOL OUTBREAK, ALL ISOLATES CLADE 3.15**						
B103	A	0	0	0	1	All co-primary cases
B104	A	0	0	0	1	
B105	A	0	Reference	0	Reference	
B106	A	0	0	0	0	
B107	A	0	0	0	1	
**TACO BELL OUTBREAK, ALL CLADE 8.30 EXCEPT EC4436-7 CLADE 8.32**						
EC4401	B	1	Reference	0	Reference	Eppinger et al., 2011b
EC4402	A	0	0	0	0	Case isolate
EC4436	A	90	Excluded	155	Excluded	Temporal outliers
EC4437	A	90	Excluded	155	Excluded	
EC4439	A	5	Excluded	17	Excluded	
EC4448	A	0	0	1	2	
EC4486	B	0	1	4	4	Eppinger et al., 2011b
**TACO JOHN, ALL ISOLATES CLADE 2.90**						
EC4501	B	1	0	6	8	Eppinger et al., 2011b
TW14588	C	0	Reference	0	Reference	
**FAIRBANK FARMS OUTBREAK, ALL ISOLATES CLADE 8.30**						
EC1845	A	0	0	0	2	Case isolate
EC1846	A	0	0	0	2	
EC1847	A	0	0	0	2	
EC1848	A	0	0	0	2	
**BATTLEGROUND LAKE OUTBREAK, ALL ISOLATES CLADE 8.32**						
B112	A	1	1	0	0	Case isolates
B113	A	0	0	0	0	
B114	A	0	Reference	0	Reference	
**COOKIE DOUGH OUTBREAK, EC1738 CLADE 6.26, EC1734-7 CLADE 8.32**						
EC1738	B	168	excluded	298	excluded	Product isolate
EC1734	B	0	5	2	5	Case isolates
EC1735	A	0	1	0	2	
EC1736	A	1	Reference	1	Reference	
EC1737	A	0	1	0	2	
**TOTINO's, PIZZA OUTBREAK ALL ISOLATES CLADE 8.31**						
EC1863	A	6	Reference	11	Reference	Case isolates
EC1864	A	9	10	10	8	
EC1866	A	6	7	12	8	
EC1868	A	6	3	6	6	
EC1869	A	7	15	9	21	
EC1870	A	10	20	9	21	
**FAIRBANK FARMS OUTBREAK, ALL ISOLATES CLADE 8.30**						
EC1849	A	0	0	0	2	Case isolate
EC1850	A	0	0	0	2	
EC1856	A	0	Reference	0	Reference	
EC1862	A	0	0	0	3	

a *This study/short reads in NCBI = A, WGS = B, assembled genomes in NCBI = C*.

**Table 3:** **Comparison of common vs. outbreak-specific reference intergenic SNPs**.

	**Isolate**	**Sequences analyzed^a^**	**SNPs in isolates, in intergenic regions**		**Comments^b^**
			**Compared to reference EC4115**	**Compared to outbreak strain**	
FS	B103	A	1	1	Reference specific allele
	B104	A	0	1	
	B105	A	0	Reference	
	B106	A	0	1	
	B107	A	0	1	
BG	B112	A	0	0	
	B113	A	0	0	
	B114	A	0	Reference	
TB	EC4401	B	1	Reference	F
	EC4402	A	0	27	
	EC4436	A	40	Excluded	
	EC4437	A	40	Excluded	
	EC4439	A	5	Excluded	
	EC4448	A	0	27	
	EC4486	B	3	31	
TJ	EC4501	B	8	16	F
	TW14588	C	1	Reference	
CD	EC1738	B	67	Excluded	F
	EC1734	B	0	4	
	EC1735	A	0	0	
	EC1736	A	0	Reference	
	EC1737	A	0	0	
TP	EC1863	A	2	Reference	High diversity
	EC1864	A	1	5	
	EC1866	A	2	6	
	EC1868	A	2	5	
	EC1869	A	10	14	
	EC1870	A	6	11	
FF	EC1845	A	0	1	Reference specific allele
	EC1846	A	0	1	
	EC1847	A	0	1	
	EC1848	A	0	1	
	EC1849	A	0	1	
	EC1850	A	0	1	
	EC1856	A	0	Reference	
	EC1862	A	0	1	

a *This study/short reads in NCBI = A, WGS = B, assembled genomes in NCBI = C*.

b *F = mixed analysis with reads missing for some strains*.

For three outbreaks (FF, FS, and BL) we identified only a single or no SNP when referenced to EC4115 (Figure 2, Tables 2, 3). A single intergenic and three nsyn mutations were identified when using an FF outbreak-specific reference strain EC1856 (Tables 2, 3). The three nsyn SNPs did not affect domain prediction in Pfam (Finn et al., 2016). B112 of the BL outbreak had a syn SNP in a tRNA-histidine ligase (#3460738) not found in any other *E. coli* O157:H7 genome deposited (nr or WGS). This SNP was identified in both instances when using EC4115 or an outbreak-specific reference (Supplemental Dataset 1). This SNP was confirmed using PCR amplicon sequencing. Using the alternative FS outbreak-specific reference one intergenic SNP in B105 and one nsyn SNP affecting the Nitrogen regulation protein NR(I) (ECH74115_RS26390) (B107 and B105) were identified (Tables 2, 3). SNP discovery predicted an outbreak-specific allele in three strains. However, these SNPs are false positives, as they could not be confirmed by PCR sequencing. The SNP (#693920) in FS strain B103 was identified as false-positive homoplastic SNP also observed in plate mate and intrahousehold strains with an allelic frequency below 0.9 (Supplemental Table 2). During SNP prediction we identified 52 SNPs in strain B103 that were not found in the other FS outbreak strains. These 52 SNPs were all located in a phage region that corresponds to the tandem integrated SP1/2 phages (Hayashi et al., 2001). The SNPs were all false-positives due to the presence of an additional phage in B103 related to a prophage from organism pro483 (NC_028943) (Supplemental Figure 3). The tail fiber proteins of these two phages were sufficiently similar to misalign reads for B103. This exemplifies the importance of SNP curation and assessment according to the genomic region in which they originate, as independent horizontal acquisition of segments can introduce epidemiologically misleading SNPs (Pettengill et al., 2014), also known as epidemiological type 2 errors of attribution.

**Figure 5:**
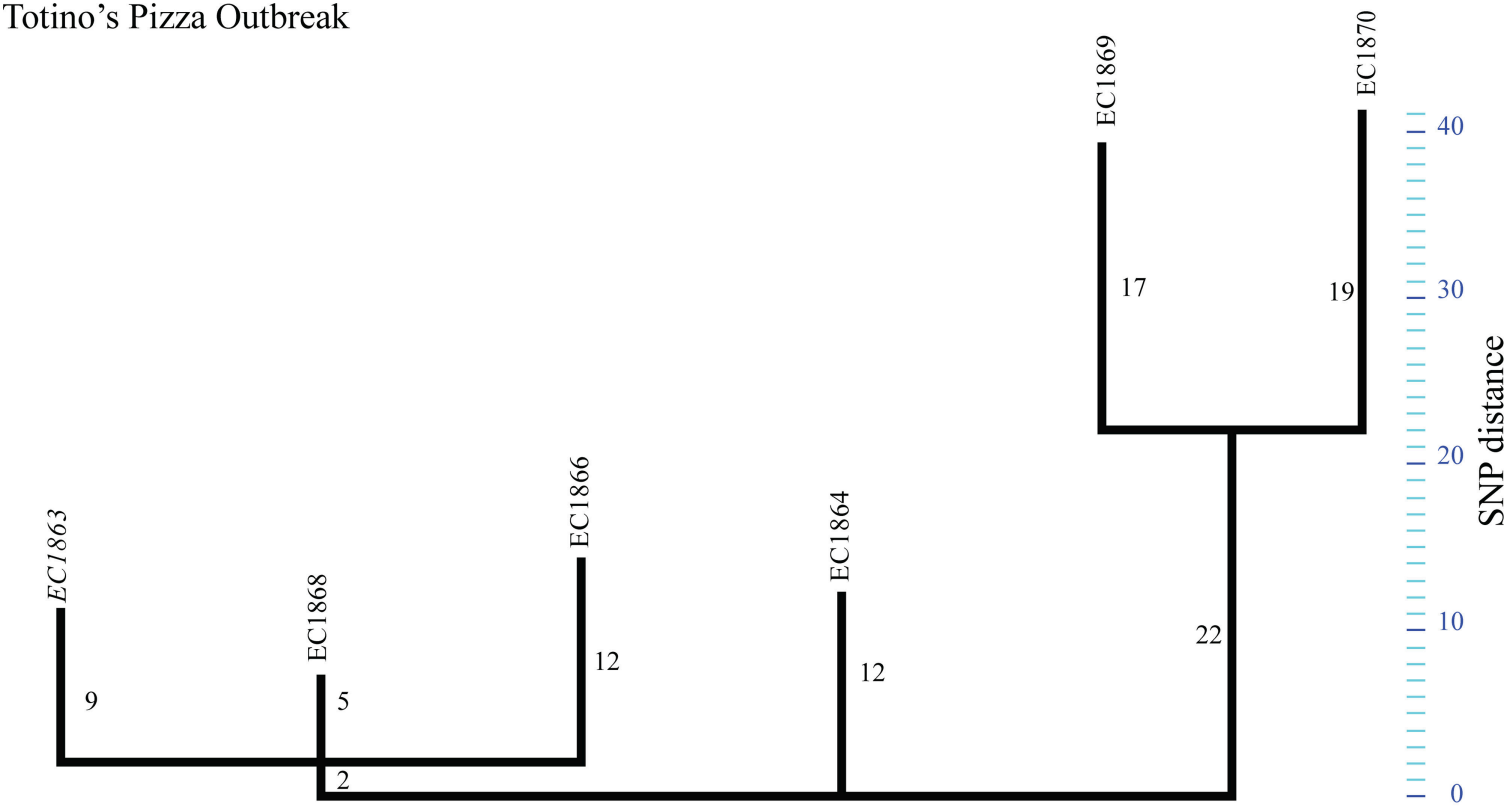
**Phylogenetic tree for Totino's Pizza outbreak strains**. Maximum parsimony phylogeny of the Totino's Pizza outbreak using reference EC1863. The tree topology shows the genotypic heterogeneity among the outbreak associated strains, forming “two” distinct phylogenetic groups.

The TP outbreak strains revealed a highly distinct SNP pattern compared to the genomic plasticity reported for other outbreaks (Figures 1, 5, Tables 2, 3). Two distinct phylogenetic clusters separated by 16 SNPs were observed. Additionally, each strain carried at least 4–17 strain-specific SNPs. Comparison to outbreak-specific reference EC1863 confirmed the relative high number of strain-specific SNPs (Tables 2, 3, Figure 5). In contrary to our observations for strain-specific SNPs in the above discussed outbreaks, these SNPs are neither concentrated in specific regions nor more frequent in intergenic than in genic regions (Tables 2, 3). The EC1869/EC1870 branch contributes roughly 60% of all SNPs (Supplemental Dataset 1). Based on the established phylogenetic topology we hypothesize that two closely related but different *E. coli* O157:H7 contaminated a common vehicle, if, indeed, all cases had the same exposure. Two-thirds of the SNPs were strain-specific, denoting a particular high diversity within this outbreak (Figures 1, 5, Tables 2, 3). Such a degree of genomic plasticity among epidemiologically linked strains has rarely been observed in *E. coli* O157:H7. Several scenarios could have led to this radial expansion: (i) the epidemiology linked cases together that actually were from different simultaneous outbreaks, (ii) the SNPs identified *in silico* are false positives and only PCR-confirmation could really confirm the true distance among the strains, (iii) the high rate of accumulated SNPs could be caused by a mutator genotype resulting in the accumulation of mutations in a short time span, (iv) the heterogeneity could be related to the protracted duration of the outbreak (3 months), vs. single, brief, single source-exposures as in the FS outbreak, or (v) heterogeneity caused by increased strain mutation rates during outbreaks as have been discussed for other enterics (Morelli et al., 2010). In support of our findings, Dallman et al. noted correlations between the length of the strain collection intervals and respective numbers of SNPs observed (Dallman et al., 2015).

The clonal nature of *E. coli* O157:H7 outbreaks was confirmed in the majority of the outbreak strains analyzed here, consistent with prior findings from SNP typing in other O157:H7 outbreaks (Turabelidze et al., 2013; Dallman et al., 2015; Holmes et al., 2015; Jenkins et al., 2015; Munns et al., 2016). We found the number of SNPs to be inversely proportional to the availability of reads. This highlights the critical importance of quality control for accurate SNP discovery by accounting for both underlying sequence quality and evolutionary context of the SNP carrying loci to curate for false-positives. In this regard, the relevance of excluding mobile regions when inferring outbreak relatedness is evidenced in the loss of at least two thirds of predicted SNPs that if considered would impair phylogenetic accuracy.

### WGS typing of plate mates recovered from human infections

In the medical praxis typically a single colony is retrieved from a primary isolation plate and sent for further molecular analysis. It is therefore not clear how much genotypic diversity exists among infecting isolates of *E. coli* O157:H7 as shed from the same individual in a single stool. To answer this question, plate-mates (pairs of colonies) were separately saved from five patients (Figure 2, Supplemental Table 1) enrolled in a multi-state study of *E. coli* O157:H7 infections (Wong et al., 2012). In the EC4115 reference-based discovery, two PM possessed the same homoplastic intergenic SNP (Figure 2, Tables 4, 5), which was not confirmed after allelic verification. When using an internal reference these strains were undistinguishable. The results are in accordance with those of Dallman et al., who reported 0–2 SNPs among same patient isolates, with most (70%) having no SNP differences at all (Dallman et al., 2015). Our results from this limited study, therefore, point toward infection with a single *E. coli* O157:H7 clone as the underlying cause for the majority of infections. We previously reported that a single laboratory passage can produce SNPs in *E. coli* O157:H7, but SNPs arise only rarely (Eppinger et al., 2011b). In the course of naturally acquired human infections, our data endorse that *E. coli* O157:H7 SNPs are exceptionally rare events.

**Table 4:** **Comparison of common vs. PM/IH-specific genic SNPs**.

	**Isolate**	**Sequences analyzed^a^**	**Synonymous SNPs in isolates, among backbone ORFs**		**Nonsynonymous SNPs in isolates, among backbone ORFs**	
			**Compared to reference EC4115**	**Compared to outbreak strain**	**Compared to reference EC4115**	**Compared to outbreak strain**
PM	B26-1	A	0	0	0	0
	B26-2	A	0	Reference	0	Reference
PM	B28-1	A	0	Reference	0	Reference
	B28-2	A	0	0	0	0
PM	B29-1	A	0	0	0	0
	B29-2	A	0	Reference	0	Reference
PM	B36-1	A	0	0	0	0
	B36-2	A	0	Reference	0	Reference
PM	B40-1	A	0	Reference	0	Reference
	B40-2	A	0	0	0	0
PM	B7-1	A	0	0	0	0
	B7-2	A	0	Reference	0	Reference
IH	B83	A	0	0	0	0
	B84	A	0	Reference	0	Reference
IH	B85	A	0	Reference	0	Reference
	B86	A	0	0	0	0
IH	B89	A	0	0	0	0
	B90	A	0	Reference	0	Reference
IH	B93	A	0	Reference	0	Reference
	B94	A	0	0	0	0
IH	B15	A	0	0	0	0
	B17	A	0	Reference	0	Reference
IH	B108	A	0	0	0	0
	B109	A	0	Reference	0	Reference

a *This study/short reads in NCBI = A, WGS = B, assembled genomes in NCBI = C*.

### WGS typing of strains from intrahousehold infections

To determine if genomic changes in infecting *E. coli* O157:H7 occur during probable intrahousehold (IH) transmission, we analyzed a cohort of six pair isolates from IH infections where onset was quite delayed between cases (Figure 2). As with the PM pairs, EC4115 based SNP discovery resulted only in false positive homoplastic intergenic SNPs (Figure 2, Tables 3, 4) that were absent in the pair-wise analysis. Dallman et al. observed similar SNP distributions in household transmission cases in the UK, with 40% having no such differences in the core genome (Dallman et al., 2015). Interestingly, two IH cases of clade type 3.15 clustered together (Figure 2). A single syn SNP was specific to the B83/B84 cluster. These cases were all from the same state and occurred in the same year, but epidemiological investigations suggest they are separate cases of IH transmissions with over 6 weeks between occurrence and 80 miles distance between the zip codes in which the cases resided. This application of WGS typing analysis can genomically link clusters that were not previously identified epidemiologically (Dallman et al., 2015).

In general the frequency of SNPs in intergenic and genic regions were similar, highlighting the random nature of SNPs identified. While there is clearly no applicable universal gold standard or criteria for outbreak ex- or inclusion in regards to SNP matrix distances, we note that a number of outbreak investigations have found between four to seven SNPs among strains with putative epidemiological links (Underwood et al., 2013; Joensen et al., 2014; Dallman et al., 2015; Holmes et al., 2015). However, these analyses are limited by only including the genic portions of the genomes and/or did not use an outbreak-specific reference for SNP discovery. This prevents identification of variations in parts of the core genome that are unique to outbreak-associated strains and not necessarily present in a distantly related closed reference strain. Moreover, only few studies use confirmatory PCR or other resequencing to validate *in silico* delineated SNPs (Eppinger et al., 2011b; Underwood et al., 2013).

### Phage profiles of clinical U.S. strains

The abundance of lambdoid phages in the EHEC O157:H7 genome hinders assembly of phage regions based on short reads alone (Eppinger et al., 2011b). Contig breaks often occur within the phage borders due to the conserved nature of structural and replication proteins and hinder individual phage-level comparisons in the fragmented phage assemblies. Therefore, we applied an alternative genome-scale strategy to comprehensively analyze *stx* allele status and losses or gains in the strain's phage ORF-omes.

Major virulence traits of *E. coli* O157:H7 are encoded on members of the mobilome that are usually stably integrated into the chromosome, such as the locus of enterocyte effacement (LEE) and *stx*-converting phages (Nataro and Kaper, 1998). Phages are key components of pathogenome evolution and their acquisitions are important events in the emergence of *E. coli* O157:H7 from an ancestral cell closely related to *E. coli* O55:H7 (Feng et al., 1998, 2007; Zhou et al., 2010). Moreover, analyses such as SNP typing that are limited to the core genome cannot provide information about the conferred pathogenic potential anchored in the mobilome. Our analysis of the 2006 SP outbreak exemplifies genomic heterogeneity that can be found in a single outbreak of O157:H7 in regards to mobilome (Eppinger et al., 2011b). Within the prophage pool (Hayashi et al., 2001) the *stx*-converting bacteriophages are of particular interest, as they encode a potent cytotoxin, Shiga toxin or Stx (Karmali et al., 2010) as direct mediator of EHEC O157:H7 disease (Krüger and Lucchesi, 2015). In *E. coli* O157:H7 the chromosomal backbone is highly conserved and genomic alterations chiefly relate to phage complement, plasticity, and respective integration sites (Shaikh and Tarr, 2003; Abu-Ali et al., 2009; Eppinger et al., 2011b, 2013; Smith et al., 2012; Yin et al., 2015). Three *stx* alleles, *stx1a, stx2a*, and *stx2c*, are found predominantly in this lineage (Scheutz et al., 2012). We used discontiguous megablast against the VirulenceFinder database to determine the toxin subtypes present in each outbreak (Joensen et al., 2014). All IH, PM, and outbreak strains carry the more potent allelic variant *stx2a* (Supplemental Table 1). In addition, all FS and TJ outbreak strains, PMs B40-1/2 and B26-1/2 and two separate IH transmission cases (B83/B84, B85/B86) carry an *stx1*-converting phage. Co-carriage of Stx2 and Stx1 can reduce Stx2a production (Serra-Moreno et al., 2008) and also attenuates end-organ toxicity of Stx2a (Donohue-Rolfe et al., 2000; Russo et al., 2016). Noteworthy, the 2006 SP outbreak associated with hypervirulence (Kulasekara et al., 2009; Abu-Ali et al., 2010) features the Stx2a/2c toxin type, with an almost complete *stx1*-converting phage occupies *yehV*. However, this atypical phage lacks *stx1*genes (Eppinger et al., 2011b). We also note that the TJ lettuce isolate TW14588 harbors two *stx2a*-converting phages integrated at *argW* and *wrbA* (Supplemental Table 1). We speculate that double *stx2a*-converting phage occupancy might also increase pathogenic potential, such as through phage dosage effects, also considering that *stx2a* is the most potent allelic subtype (Tesh et al., 1993; Tesh, 2010; Fogg et al., 2012). We note that this information cannot be gathered by PCR-based Stx-subtyping (Scheutz et al., 2012), as this approach does not determine copy number, highlighting the increased resolution obtained by WGS in regards to the pathogenic potential of the outbreak (Holmes et al., 2015). All other outbreaks except BL possess *stx2c*-converting bacteriophages. The interplay between these two *stx2*-converting phage types is not known, although both variants have been linked to HUS (Friedrich et al., 2002; Persson et al., 2007). We observed only two variations in the *stx2* allelic profiles in the CD and TP outbreaks (Supplemental Table 1). The non-clinical CD outlier EC1738 collected in the production plant is distinguished by lack of the *stx2a* allele. TP strain EC1870 lacks the *stx2c* allele that is present in all other TP outbreak strains (Supplemental Table 1).

**Figure 6:**
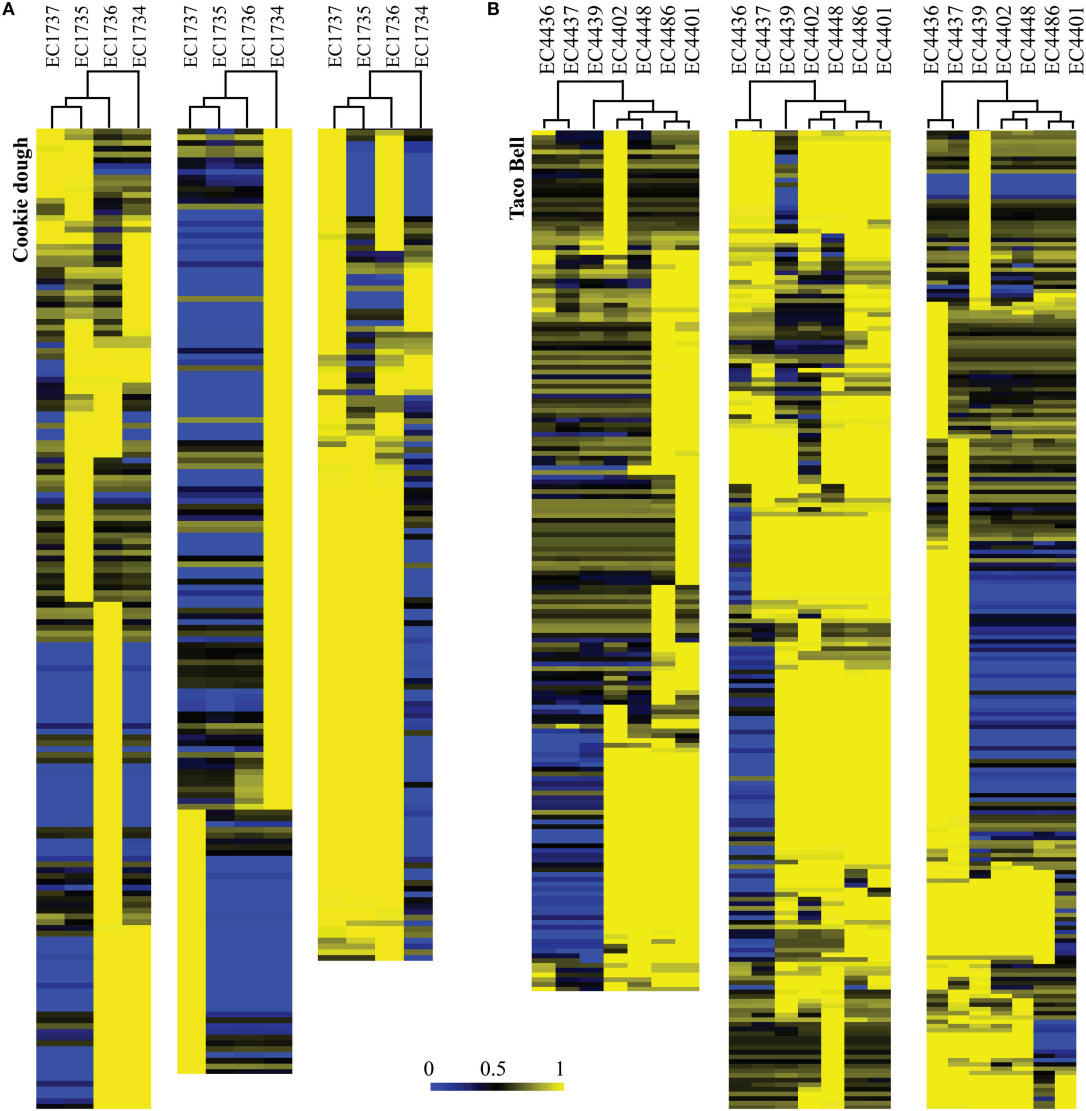
**Hierarchical clustering of the computed phage variome for Cookie dough (A) and Taco Bell (B) outbreaks**. Phage inventories within the respective outbreak clusters compared by LS-BSR and variome differences are presented in this heatmap. BSR values range from 0 (blue, absent) to 1 (yellow, identical). **(A)** Strain EC1734 contributed the majority of varying regions within the CD phage complement. **(B)** The tree topology derived from SNP typing for the TB outbreak (Figure 2) is in accordance with the variome clustering. Most of the variation was introduced by phages prevalent in the three temporal outlier strains EC4436, EC4437, and EC4439.

On average, the phage complement in the strains represents 14% of predicted coding regions in the genomes, in accordance with other studies (Asadulghani et al., 2009; Smith et al., 2012). As expected when considering the close relation between outbreak strains (Figure 2), the variability in phage-borne ORFs was low (5%) (Supplemental Figure 1; Table 3). This small variability represents the noise caused by clustering of related proteins into centroids in the LS-BSR analysis rather than differences in phage regions. The TB associated strains had more variability (11.2%) (Supplemental Table 3), due to inclusion of the temporal outliers EC4436, EC4437, and EC4439 (Figure 6). Variome analysis highlighted phage regions that were unique to each temporal outlier group, resulting in the same clustering as in the SNP-based analysis (Figures 2, 6). The CD outbreak also had greater variability (16.5%) despite the exclusion of outlier EC1738 (Supplemental Table 3), likely attributable to differences in fragmentation of the analyzed genomes. We observed a correlation of quality of PHAST prediction with size of contigs and reduced genome fragmentation. Closed genomes and genomes with larger contigs had up to 20% more predicted phage regions that also served to increase the noise, compared to more fragmented genomes (Supplemental Table 1).

The identified phage complements of the FS isolates were highly similar. However, we found phage sequences unique to strain B103 (Supplemental Figure 2A). Discontiguous megablast of the phage region (Buhler, 2001; Ma et al., 2002) against closed bacteriophages identified *Escherichia* phage pro483 (KR073661), originally isolated from an avian pathogenic *E. coli* DE048. This prophage was previously described in SP strains (Eppinger et al., 2011b) and a supershedder strain SS17 (Cote et al., 2015). Unlike the *yegQ* insertion in SP outbreak strains (Eppinger et al., 2011b), this phage disrupts the colicin immunity protein (WP_001303895) in strain B103. Using the phage pro483 (KR073661) as a genomic anchor for the B103 draft contigs we recovered 86% of the phage genome with 97% identity. We further identified a 12 bp (ACCAATAACTGA) repeat at both ends of the phage borders, indicative of the phage integration mechanism (Campbell, 1992). The SP outbreak strain EC4115 however features an 18 bp repeat (Eppinger et al., 2011b). The genomic architecture is syntenic and largely conserved throughout the phage genome, except for insertion or deletion introduced by an exonuclease (ECH74115_RS15445) and a hypothetical protein (ECH74115_RS15450) only present in EC4115 (Supplemental Figure 3).

Acquisition or loss of phages secondary to recombination events during the course of an outbreak creates interstrain plasticity. Thus, analysis of a single archetypical outbreak strain might underestimate the mobilome and core chromosome plasticity (Eppinger and Cebula, 2015). Comprehensive analyses did not reveal significant differences in phage content of the BL and FF outbreak clusters (Figure 2) to further distinguish these clonal outbreaks featuring only one and four SNPs per outbreak cluster, respectively (Supplemental Figures 2B,C). In contrast, the TP outbreak strains displayed a higher degree of mobilome plasticity (Supplemental Figure 2D), in line with the higher number of predicted SNPs (#98) for this outbreak (Figure 2, Supplemental Dataset 1).

### Plasmid prevalence in clinical *E. coli* O157:H7 strains

The *E. coli* O157:H7 lineage is distinguished from other serotypes by the presence of the large virulence plasmid pO157 (Burland et al., 1998). For this serotype, additional plasmids have been occasionally characterized at sequence level (Makino et al., 1998; Eppinger et al., 2011b) or by plasmid profiling (Ostroff et al., 1989; Meng et al., 1995). To facilitate plasmid discovery and survey we reassembled the genomes with SPAdes (Bankevich et al., 2012). Even though deposited genomes from 454 and Illumina Celera hybrid assemblies (Denisov et al., 2008) had fewer contigs compared to SPAdes assemblies from Illumina reads only (Supplemental Table 1), reassembly typically produced longer contigs, in particular for plasmid-originating regions. If Illumina reads only were processed, the SPAdes assemblies clearly outperformed NCBI deposited Velvet assemblies in regards to sensitivity for plasmid prediction (Supplemental Table 1). We queried plasmid sequences against the NCBI nr plasmid database using discontiguous megablast (Buhler, 2001; Ma et al., 2002).

Using this approach we discovered five plasmids at the sequence level (p78, p34, p55, p63, and p39) that have not been previously described in deposited *E. coli* O157:H7 genomes. Among these is a homolog of a 37 kb conjugal transfer pEC4115, referred to as p36, originally described in the SP outbreak strains (Eppinger et al., 2011b). We found the TB and TP outbreaks to be most diverse in regards to plasmid carriage (Figure 2). The TB associated strains contained three distinct plasmid profiles, which correlated with the clustering from the core genome SNP discovery (Figure 2).

**Figure 7:**
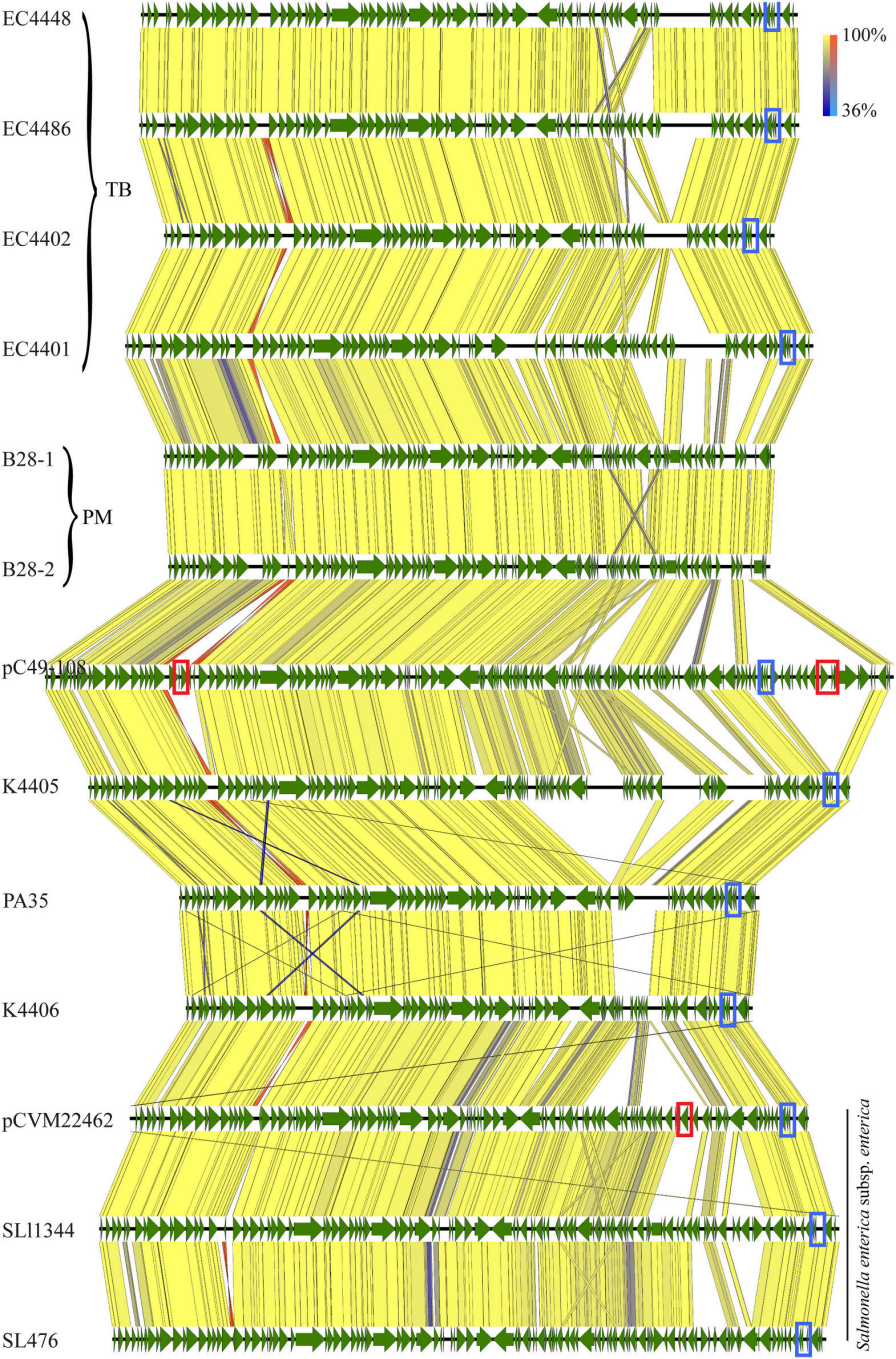
**Alignment of plasmid p78 and homologs**. Plasmid architecture and gene inventories were compared by tblastx, and respective annotations were mapped in Geneious vR9 (CDS, green). The plasmid architecture was highly conserved with a high identity level throughout the entire length of the plasmid [100% identity yellow (inverted fragment, orange), 36% identity blue (inverted fragment light blue)]. Plasmid p78 homologs were widespread, such as in TB outbreak associated strains, B28 plate mates, other *E. coli* O157:H7, and *S. enterica*. We found a locus for a RelB/E tox/antitoxin system present in all plasmids, with the exception of PM strains B28 (blue box). Resistance loci are highlighted in red boxes.

Plasmid p78, the largest plasmid, shows homology to the conjugative IncI1 group *E. coli* plasmid pC49-108 and *Salmonella enterica* plasmids (Fricke et al., 2011; Kröger et al., 2012; Wang et al., 2014a). p78 varied in length, from 78 to 88 kb in clade 8 strains (Figure 7). The related plasmid pC49-108 carries multiple antibiotic resistance genes (Wang et al., 2014a), including a beta-lactamase (*bla*_CTX−M−1_) (Wang et al., 2014a), dihydrofolate reductase (*dfrA17*) and aminoglycoside adenylyltransferase (*aadA5*) found both adjacent to a class 1 integron (Wang et al., 2014a). In similarity to the *bla*_CTX−M−1_ located next to a mobile element (ISECp1), we found another class C beta-lactamase gene in *S. enterica* CVM 22462, again found next to a mobile transposase locus. We speculate that colocalization to mobile elements might affect locus stability and explains the scattered prevalence of these resistances in the plasmid homologs (Wang et al., 2014b) (Figure 7).

Resistance to antibiotics has been observed in *E. coli* O157:H7, but the genetic basis remains largely unknown (Meng et al., 1998). We previously linked multi drug resistance (MDR), a rare occurrence in *E. coli* O157:H7, to phage-borne antibiotic resistance loci (Eppinger et al., 2011a). Strain EC4402, part of the core TB outbreak cluster, was identified as a MDR isolate (Figure 2). This strain displays elevated MICs for several cephalosporins and aminoglycosides, sulfisoxazole and nalidixic acid (quinolone). However, our *in silico* analysis with ResFinder (Kleinheinz et al., 2014) did not reveal any potential underlying resistance loci. Here we note that resistance phenotypes can be conferred by loci not previously linked to antibiotic resistance (Gibson et al., 2016). We speculate that the resistance loci might have been either lost from the original p78, or were an integral part of other MDR plasmids lost during laboratory cultivation prior to the sequencing of EC4402. Alternatively, the antibiotic resistance might be conferred by yet unknown loci not represented in queried resistance databases.

Plasmid p36 was highly homologous to other conjugal transfer plasmids, such as *S. enterica* plasmid pCFSAN000111_01 (NZ_CP007599) (Timme et al., 2012) and pEC4115 (Eppinger et al., 2011b) (Supplemental Figure 4). While p78 was found solely in clade 8, p36 seems to be more widespread (Cote et al., 2015), and present in non-O157:H7 *E. coli* serotypes. Co-carriage of a p78-p36 combination was also found in clade 8 strains K4405 and K4406 (Figure 7, Supplemental Figure 4). The TB outlier strain EC4439 lacks both p78 and p36, but carries a p55 plasmid with high homology to *Klebsiella pneumoniae* pDMC1097-77.775 kb (87% coverage, 99% identity) (Wright et al., 2014) (Supplemental Figure 5). This IncI2 group plasmid carries multiple resistances, which are absent in the *E. coli* plasmid homologs (Supplemental Figure 5). Interestingly, this plasmid is also present in IH strain B86, but absent from strain B85, either because of independent acquisition or secondary loss in B85, respectively (Figure 2). Our findings on plasmid prevalence are in accordance with those of Dallman et al. who showed that epidemiologically linked strains can vary largely in their plasmid inventory (Holmes et al., 2015).

The IH strains B89 and B90 harbor p34 (Supplemental Figure 6), which is related to *E. coli* pVR50, an F-like conjugative MDR plasmid (Beatson et al., 2015). While the overall plasmid backbone is conserved, p34 lacks any resistance loci (Supplemental Figure 6). The TP strains also possess strain-specific plasmids: p36 in EC1870, p63 in EC1863, and p38 in EC1868 (Figure 2). Plasmid p63 has partial homology to pO26-Vir, an IncK group plasmid, a mosaic of multiple plasmids (Fratamico et al., 2011). In 1863 (p63) we found homologous loci for conjugal transfer and type IV pili (Supplemental Figure 7), which have been implicated in cell adherence and biofilm formation (Dudley et al., 2006), and notably, these phenotypes are strain-dependent in *E. coli* O157:H7 (Vogeleer et al., 2014). A 39 kb plasmid fragment in EC1868 (p39) was found to be homologous to a 87 kb INcFII plasmid from *E. coli* (pGUE-NDM) (Bonnin et al., 2012) (Figure 2).

The observed variability in plasmid type and prevalence in the individual strains clearly highlights genomic plasticity that exists even among closely related isolates of the same origin and can be utilized for strain attribution (Eppinger et al., 2011b). The identified heterogeneity between the mobilome of outbreak strains stresses the importance of studying a number of isolates from the same outbreak instead of using archetypal outbreak strains, which as shown might not fully reflect the plasticity in the outbreak population (Eppinger and Cebula, 2015). Interestingly, all the above described *E. coli* O157:H7 plasmids lack antibiotic resistance loci, even though our plasmid survey found widespread resistances among homologous plasmids in other serotypes and species.

## Conclusion

While some of these clinical isolates have been studied previously using molecular epidemiology techniques (Samadpour et al., 2002), we have for the first time applied whole genomics epidemiology approaches (Eppinger et al., 2011b). Through these high resolution methods we established a detailed understanding of the genomic heterogeneity found among the studied *E. coli* O157:H7 outbreak populations from the U.S. The gathered phylogenomic data were critical to define the genetic relatedness of individual strains in the context of outbreak etiology and phylogenetic positions in the broader model of *E. coli* O157:H7 evolution and epidemiology. In this study, we detected previously unnoted polymorphic genome features in the core and mobile genome, such as an array of new plasmids not previously associated with this lineage. The cataloged polymorphic signatures aided in strain attribution and allowed us to precisely define the outbreak boundaries. This allowed us to discern the distinct phylogenetic boundaries of the studied EHEC strains when placed into a larger phylogenomic framework of *E. coli* O157:H7 from North America (Figure 2) assessing both core and mobilome (Figures 6, 7) (Eppinger and Cebula, 2015). The developed WGS typing approach provided us with the necessary resolution power to study the individual dynamics in highly clonal outbreaks (Morelli et al., 2010; Eppinger et al., 2011b, 2014; Hasan et al., 2012; Berenger et al., 2015; Holmes et al., 2015; Jenkins et al., 2015).

While the majority of outbreaks were caused by pathogens that form tight clonal clusters, one outbreak (“Totino's Pizza”) was associated with isolates showing considerable genomic heterogeneity (Figures 2, 5). Apparent SNPs in other outbreaks are associated with a paucity of reads for quality control, falsely increasing the diversity among the outbreak isolates. Since outbreaks can have high economic impacts, such as nationwide recalls of contaminated product, multiple samples from the same outbreak should be concomitantly sequenced instead of using archetypal outbreak strains to provide strong evidence for inclusion or exclusion, strain and source attribution (Eppinger et al., 2010, 2014; Morelli et al., 2010; Hasan et al., 2012). Additionally, these high resolution approaches allow for the discovery of emerging pathotypes, and, potentially, to better assess the pathogenic potential of individual bacterial clones (Berenger et al., 2015; Klemm and Dougan, 2016). Expanding these sequence-based analyses to the publicly available EHEC sequence pool will improve public health response in the event of an outbreak allowing timely and informed countermeasures. Canonical SNPs can be implemented in efficient typing assays offering robust phylogenetic signals for outbreak exclusion/inclusion that surpass classical technologies (Riordan et al., 2008; Elhadidy et al., 2015; Rusconi and Eppinger, 2016).

**Table 5:** **Comparison of common vs. PM/IH-specific intergenic SNPs**.

	**Isolate**	**Sequences analyzed^a^**	**SNPs in isolates, in intergenic regions**		**Comments^b^**
			**Compared to reference EC4115**	**Compared to outbreak strain**	
PM	B26-1	A	0	0	
	B26-2	A	0	Reference	
PM	B28-1	A	1	Reference	Homoplastic FP
	B28-2	A	0	0	
PM	B29-1	A	0	0	
	B29-2	A	0	Reference	
PM	B36-1	A	1	0	Homoplastic FP
	B36-2	A	0	Reference	
PM	B40-1	A	0	Reference	
	B40-2	A	0	0	
PM	B7-1	A	0	0	
	B7-2	A	0	Reference	
IH	B83	A	1	0	Homoplastic FP
	B84	A	0	Reference	
IH	B85	A	0	Reference	Homoplastic FP
	B86	A	1	0	
IH	B89	A	0	0	
	B90	A	0	Reference	
IH	B93	A	0	Reference	
	B94	A	0	0	
IH	B15	A	0	0	Homoplastic FP
	B17	A	1	Reference	
IH	B108	A	0	0	
	B109	A	0	Reference	

a *This study/short reads in NCBI = A, WGS = B, assembled genomes in NCBI = C*.

b *False positive = FP*.

Our study strongly endorses that quality of SNPs and choice of an appropriate reference strain in WGST approaches are equally critical to achieve phylogenetic resolution and accuracy (Table 6). Here we also demonstrate that in order to avoid type 2 error of attribution, the quality of SNP data obtained from WGS approaches is crucial (Table 6). For read-based discovery approaches we would like to emphasize the importance of SRA data availability, which is not only foundational to determine coverage and quality of detected SNP positions, but also to optimize assembly quality should assemblers with improved algorithms become available (Supplemental Table 1). SNP discovery with an appropriate outbreak-specific reference strain is critical for reference based WGS typing. To fully assess the genomic plasticity, the reference should be phylogenetically related and not too distant to the strains of interest, as evidenced by the resolution power gained using a within outbreak reference (Figures 2, 4, 5). By extending our analysis to the mobilome, we detected plasticity among clonal strains in phage and plasmid content describing novel plasmids not previously associated with *E. coli* O157:H7. We also would like to stress the importance of publicly available strain associated clinical, environmental, and epidemiological metadata concomitantly to the genomic data as prerequisite for informed source attribution (Table 6) (Eppinger and Cebula, 2015). We anticipate that NGS long-read technology, such as contemporary SMRT technology (English et al., 2012), or other platforms under development (Feng et al., 2015; Rhoads and Au, 2015) will tremendously benefit WGS typing strategies as it pertains to the highly homogenous *E. coli* O157:H7 lineage (Zhang et al., 2006, 2007; Eppinger et al., 2011b). In particular, long-read technologies will produce (near) closed genomes and thus allow to accurately determine the *stx*-virulence status by defining not only *stx* allele type, but also *stx*-converting phage combination, plasticity, and location, all factors that have been associated with alterations in Stx-production as direct mediator of EHEC disease (Ogura et al., 2015; Toro et al., 2015; Yin et al., 2015).

**Table 6:** **Proposed criteria and practices for SNP-based epidemiological outbreak inclusion or exclusion**.

**Ideal case**	
**PRIMARY CRITERIA**	
Highly credible exposure	Point source (clustered in time and space), pathogen isolated from incriminated vehicle
High quality sequencing	Contig numbers should be comparable to previous assemblies performed with the same assembly method, minimum coverage required will depend on technology used
Mobilome exclusion	Data focus on the most immutable part of the genome, most commonly the genome backbone
SNP validation	Second method, PCR confirmation or re-sequencing to validate mutations in discriminatory SNPs
**SECONDARY CRITERIA**	
Collection of multiple isolates from cases for accurate attribution	The larger the sample that demonstrates homogeneity, the greater the likelihood of a common source
	If a common PFGE/MLVA type is present in the same region, confirmation that allelic differences exist between outbreak strain and background non-outbreak strains
SNP calling	Reference and outbreak based SNP comparisons
	Allelic frequency >=0.9
	Base and mapping quality control
Novel SNP	If a tolerance is set at >0 for SNPs as a cut point for assigning isolates as being from the same source, a SNP that is not in the database (i.e., apparently *de novo*) would be given more credence than one that has been described previously, and which probably represents a different lineage or located in a known polymorphic gene, such as *rpoS*
**QUESTIONABLE CRITERIA**	
Variances in the complementary mobilome, such as presence of plasmids and phages	Loci of likely mobile origin are not reliable as a differentiating trait for epidemiologic purposes
**BEST PRACTICES IN REPORTING RESULTS**	
Report exclusion criteria	A list of loci and regions that were excluded from SNP discovery
Provide list of SNP loci and alleles	Provide information on location of SNP, product, resulting codon change
Provide reads	Deposition of sequences and strain associated metadata in public repositories

Our data provide insight into the maximum number of permissible SNPs two strains can have and still designate them of the same origin. In prior work, we found no SNPs between 24 isolates of the same point-source cluster, focusing on backbone ORFs (Turabelidze et al., 2013). Dallman et al. and others tolerated up to 4 SNPs in the core genome before assigning two isolates to different sources (Underwood et al., 2013; Joensen et al., 2014; Dallman et al., 2015; Holmes et al., 2015). We found one *bona fide* SNP in the course of a single point-source, short-term outbreak. Since no gold standards have yet been accepted for *E. coli* O157:H7 WGS typing we propose the following criteria (Table 6) for inclusion (presumably of same source) vs. exclusion (presumably of different source) of investigated isolates: (i) High-quality whole genome sequence fortified with extensive epidemiological outbreak data, (ii) genome-scale SNP discovery based on high quality sequencing with reference, (iii) exclusion of mobilome and repeats (to reduce epidemiological noise), followed by (iv) PCR-confirmation of eventual SNPs for definitive in-/exclusion, and (v) mobilome discovery which can significantly contribute to the genomic plasticity. Moreover, for cases that are quite dispersed in time and space, there should be greater stringency in assigning “like” status to two strains that are even differentiated by a single SNP. When outbreaks occur, there are often large product liability issues at stake, and considerable obligation on disease control authorities to identify such clusters and molecular typing serves an increasingly important role. Therefore, diligence should be exercised in choice of sequence-based typing protocols, and in their analysis.

Finally, while we eagerly anticipate the introduction of sequence-based pathogen typing as a public health and disease prevention tool (Sadiq et al., 2014; Eppinger and Cebula, 2015), we share the concern of Osterholm (2015), who stresses that this powerful technology be employed as an adjunct to, and not a replacement for, case interviewing (descriptive epidemiology) and environmental investigations. Also, we are entering an era of non-culture diagnosis of enteric infections, including those caused by *E. coli* O157:H7 (Schatz and Phillippy, 2012; Klemm and Dougan, 2016). The high resolution data presented in this article would not have been possible without classic diagnostic microbiology laboratory recovery of the pathogen of interest. We hope that resources will be devoted to recovering these agents from submitted specimens, so as to complement case investigation by local healthy jurisdictions.

## Accession number

The sequence data sets analyzed in this study have been retrieved from the short read archive (SRA) and whole genome shotgun repository at NCBI. Accession numbers for the genomes are provided in Supplemental Table 1.

## Author contributions

Conceived and designed the experiments: ME. Analyzed the data: BR, FS, SK, MM, PT, ME. Contributed reagents/materials/analysis tools: MM, PT, ME. Wrote the paper: BR, SK, MM, PT, ME.

## Funding

The study was supported by the National Institute of Allergy and Infectious Diseases, National Institute of Health, Department of Health and Human Services under contract SC2AI120941, the US Department of Homeland Security under contract 2014-ST-062-000058, the Department of Biology, the South Texas Center for Emerging Infectious Diseases (STCEID) at the University of Texas at San Antonio, and the High Performance Computing Center (HPC) under contract 2G12RR013646-12. Strain collection and archiving was funded by R01DK52081 and P30DK052574 for the Biobank Core. BR was supported in part by the Swiss National Science Foundation Early Postdoc Mobility Fellowship (P2LAP3-151770). FS was supported in part by the South Texas Center for Emerging Infectious Diseases (STCEID) and through an UTSA Teaching Fellowship (UTF).

### Conflict of interest statement

The authors declare that the research was conducted in the absence of any commercial or financial relationships that could be construed as a potential conflict of interest.
