# Supplementary material for: Whole Genome Sequencing for Genomics-Guided Investigations of Escherichia coli O157:H7 Outbreaks
Source: Front Microbiol. 2016 Jun 30;7:985. doi: 10.3389/fmicb.2016.00985 (PMC4928038; doi:10.3389/fmicb.2016.00985)
Supplement: Supplementary file 3 [file Table3.docx]

**SUPPLEMENTAL TABLE 3 | Phage Inventory Statistics**

| **Plate mates** | **CDS** | **Variome** | **%** |
| --- | --- | --- | --- |
| B7 | 1415 | 72 | 5.1 |
| B26* | 1357 | 70 | 5.2 |
| B28 | 1327 | 78 | 5.9 |
| B29 | 1288 | 62 | 4.8 |
| B36 | 1314 | 75 | 5.7 |
| B40 | 1475 | 74 | 5.0 |
| **AVERAGE** |  |  | 5.3 |
| **Intra household** | **CDS** | **Variome** | **%** |
| B15 / B17 | 1477 | 87 | 5.9 |
| B83 / B84 | 1391 | 77 | 5.5 |
| B85 / B86 | 1378 | 70 | 5.1 |
| B89 / B90 | 1398 | 83 | 5.9 |
| B93 / B94 | 1442 | 75 | 5.2 |
| B108 / B109 | 1369 | 92 | 6.7 |
| **AVERAGE** |  |  | 5.7 |
| **Outbreaks** | **CDS** | **Variome** | **%** |
| Taco Bell | 5535 | 620 | **11.2** |
| Finley School District | 3212 | 236 | 7.3 |
| Taco John | 2314 | 47 | 2.0 |
| Fairbanks Farm | 5323 | 299 | 5.6 |
| Battleground Lake | 2136 | 153 | 7.2 |
| Totino's Pizza | 4198 | 401 | 9.6 |
| Cookiedough | 2900 | 478 | **16.5** |
| **AVERAGE** |  |  | 8.5 |
|  |  |  |  |
| * temporal isolates |  |  |  |
