# Supplementary figures and images for: Whole Genome Sequencing for Genomics-Guided Investigations of Escherichia coli O157:H7 Outbreaks
Source: Front Microbiol. 2016 Jun 30;7:985. doi: 10.3389/fmicb.2016.00985 (PMC4928038; doi:10.3389/fmicb.2016.00985)

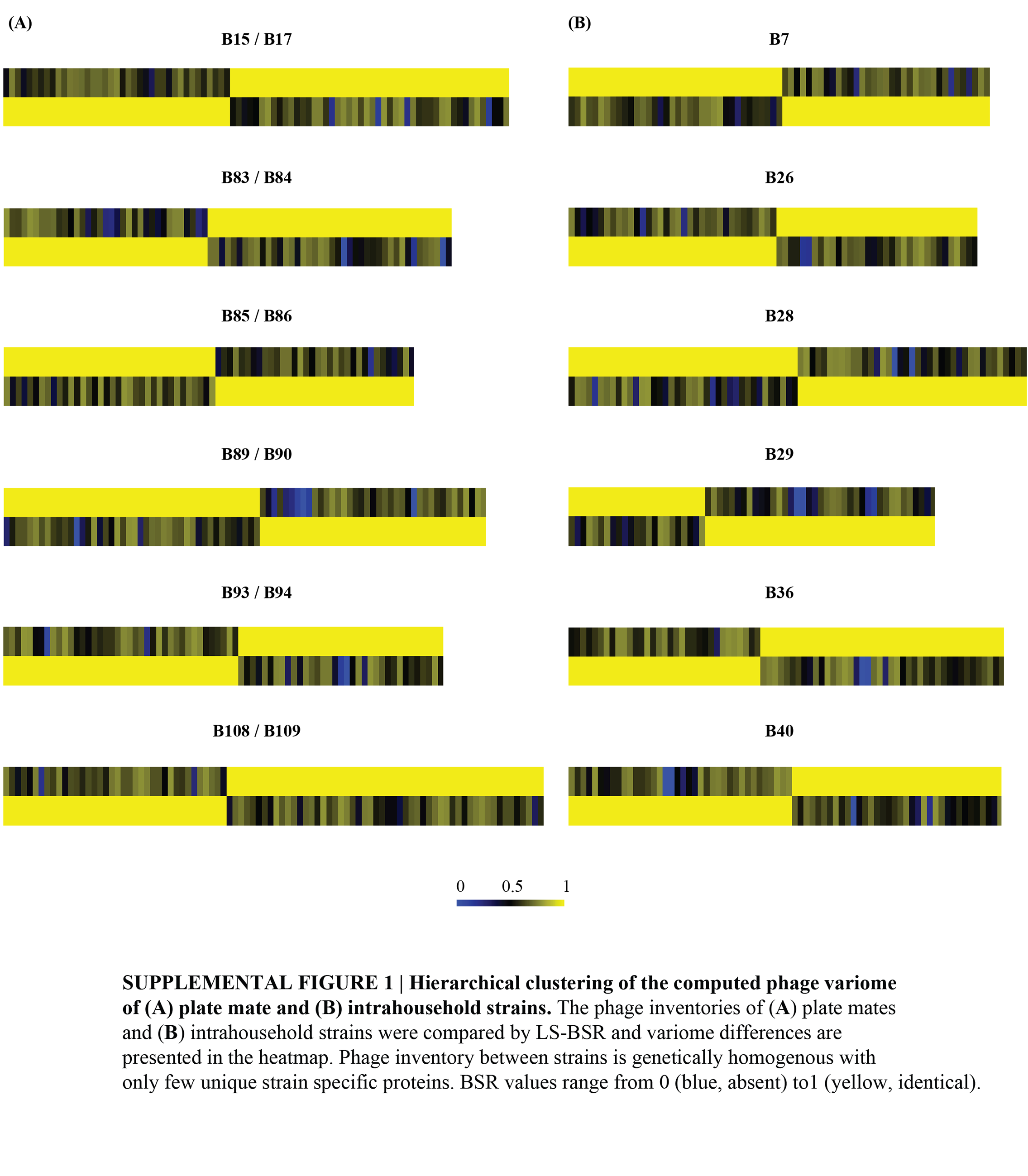

Supplement: Supplementary file 5 [file Image1.TIF]

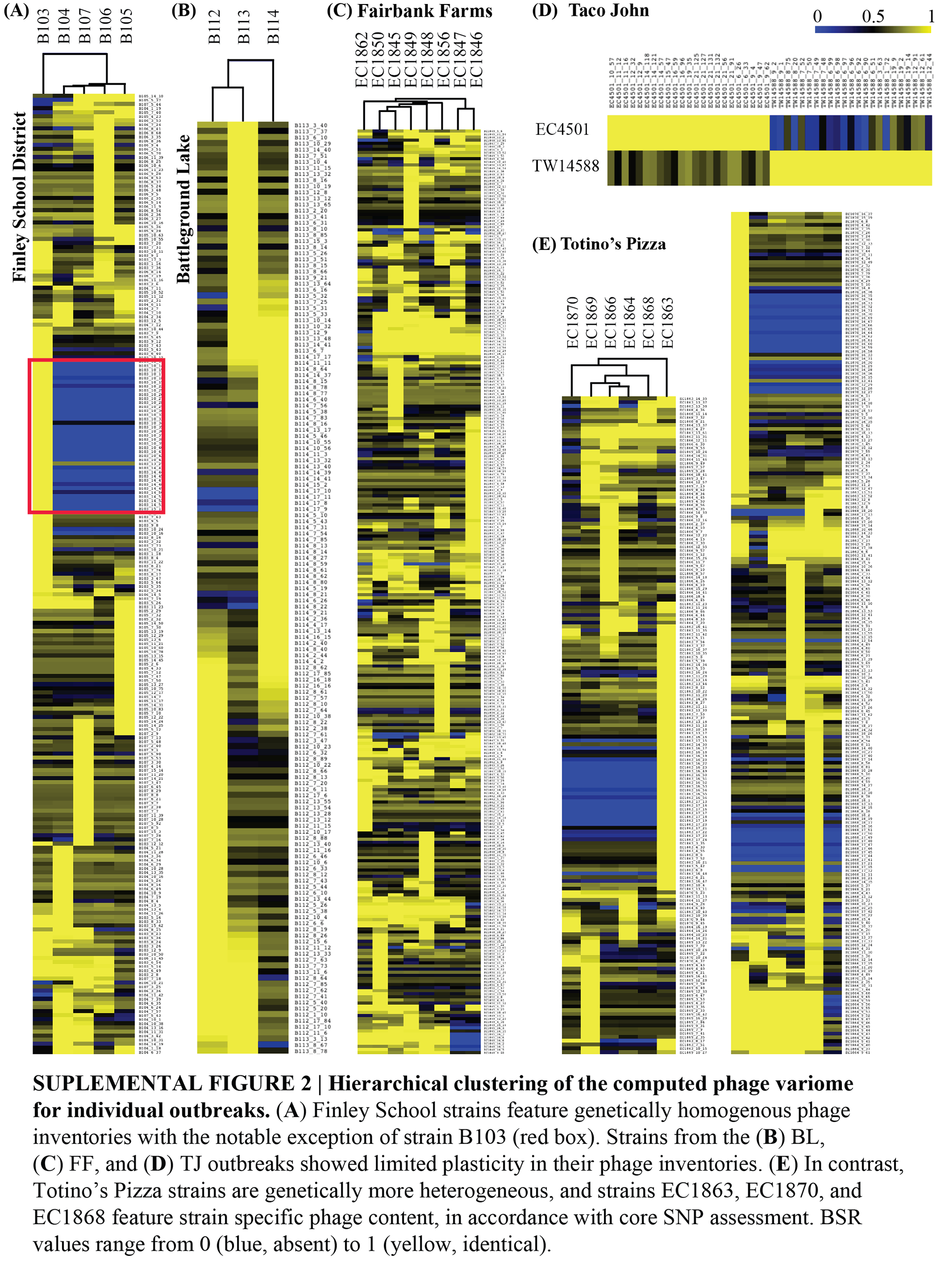

Supplement: Supplementary file 6 [file Image2.TIF]

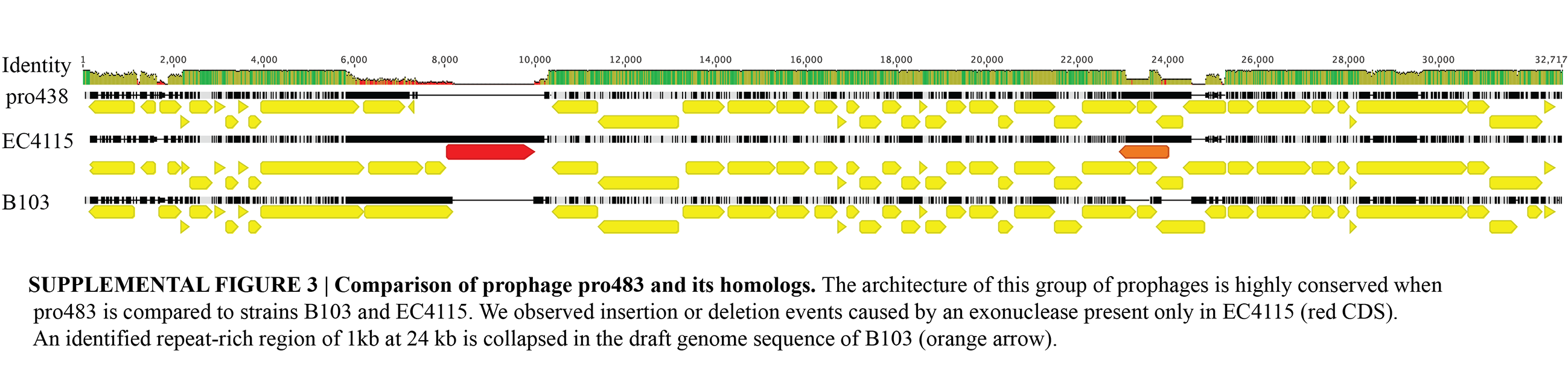

Supplement: Supplementary file 7 [file Image3.TIF]

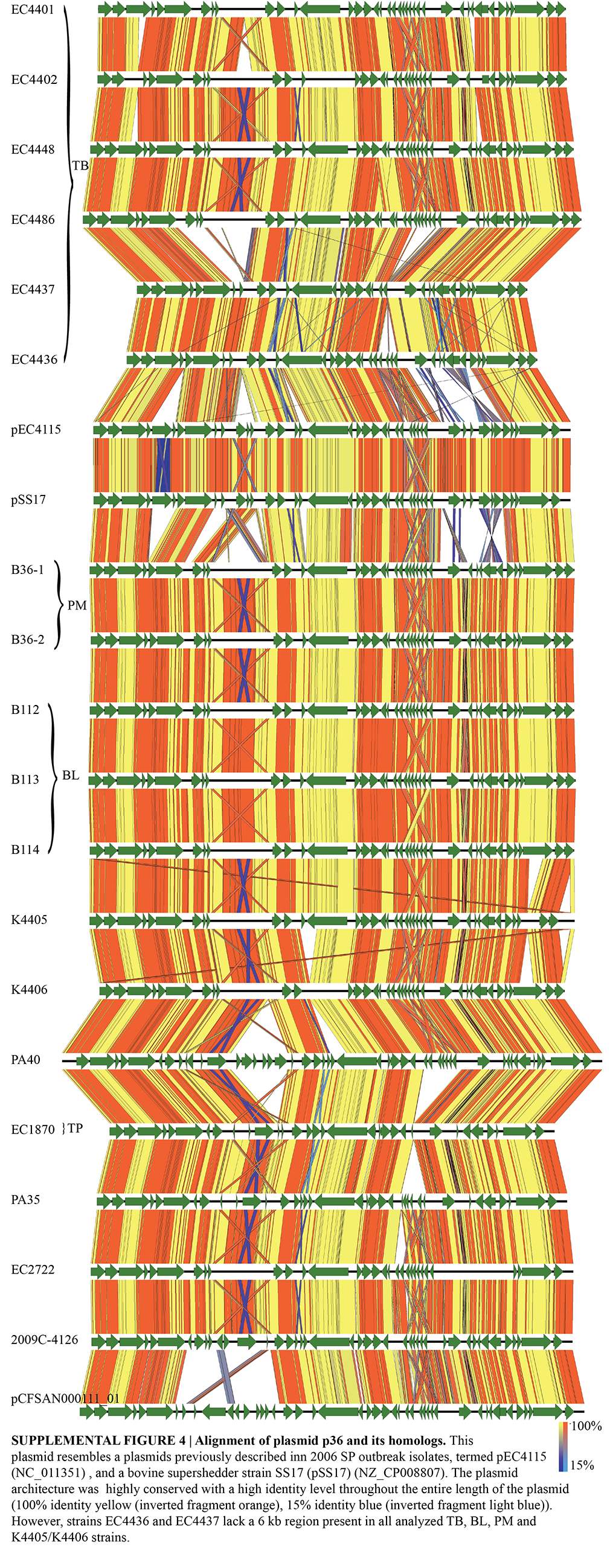

Supplement: Supplementary file 8 [file Image4.TIF]

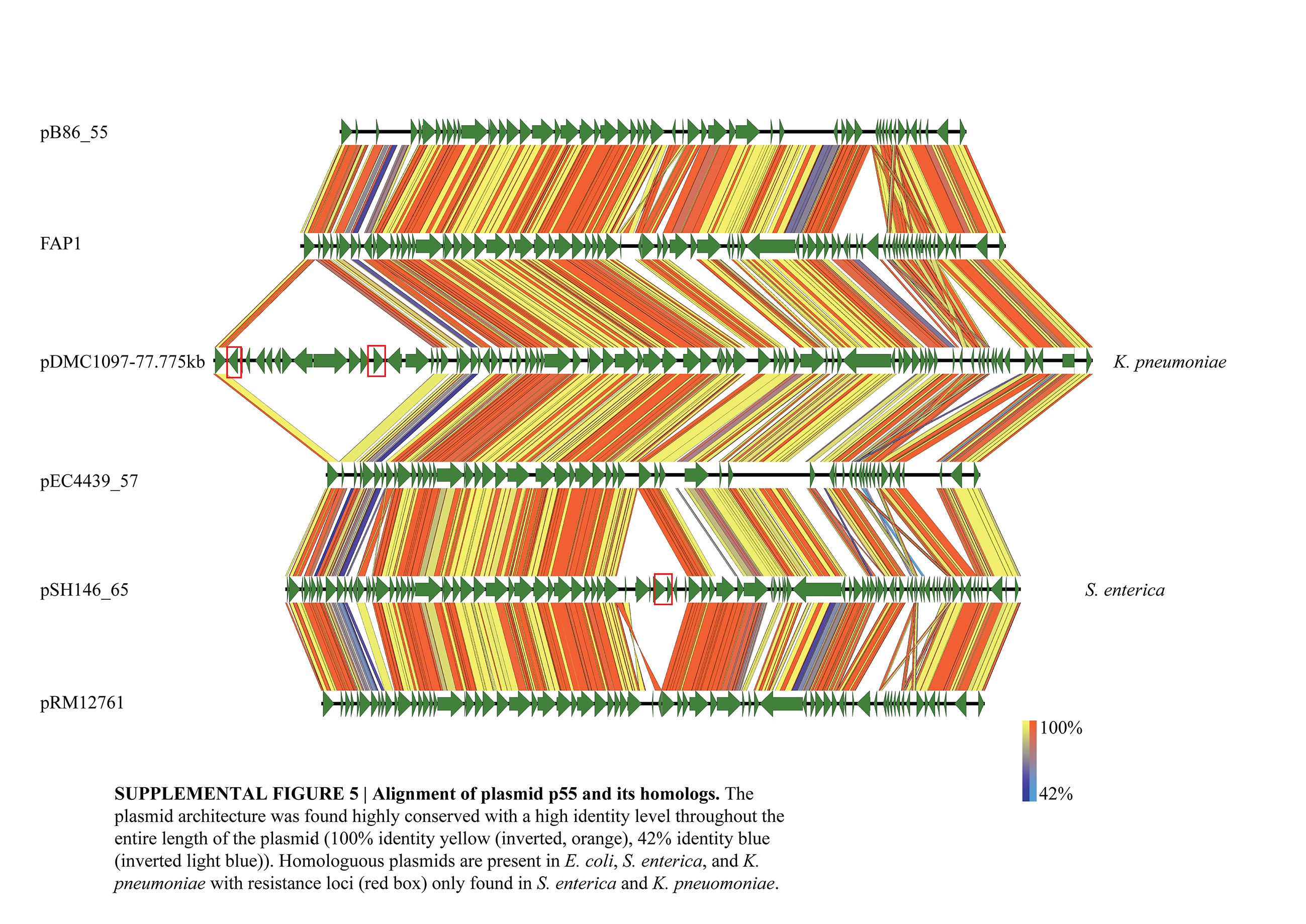

Supplement: Supplementary file 9 [file Image5.TIF]

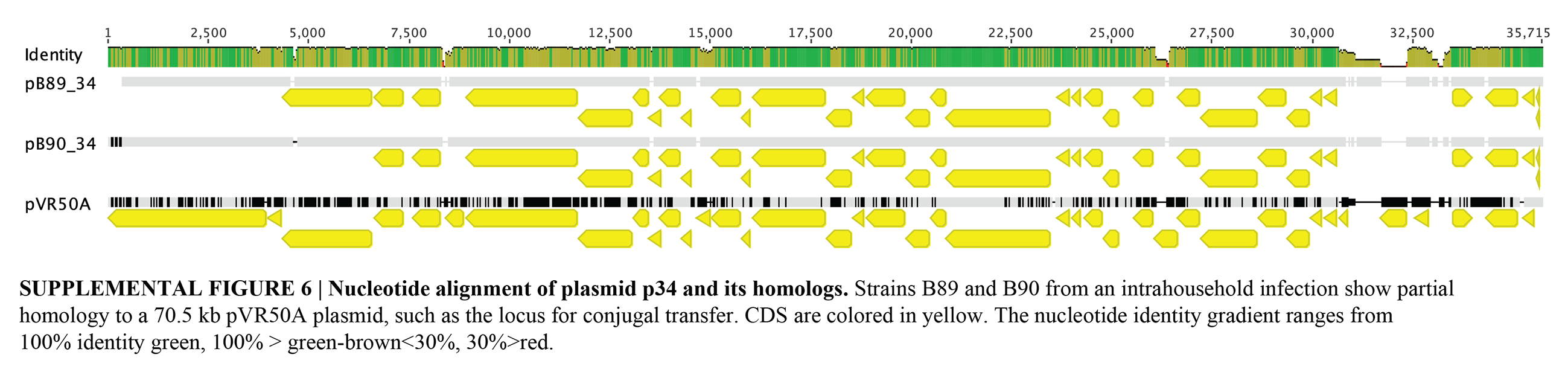

Supplement: Supplementary file 10 [file Image6.TIF]

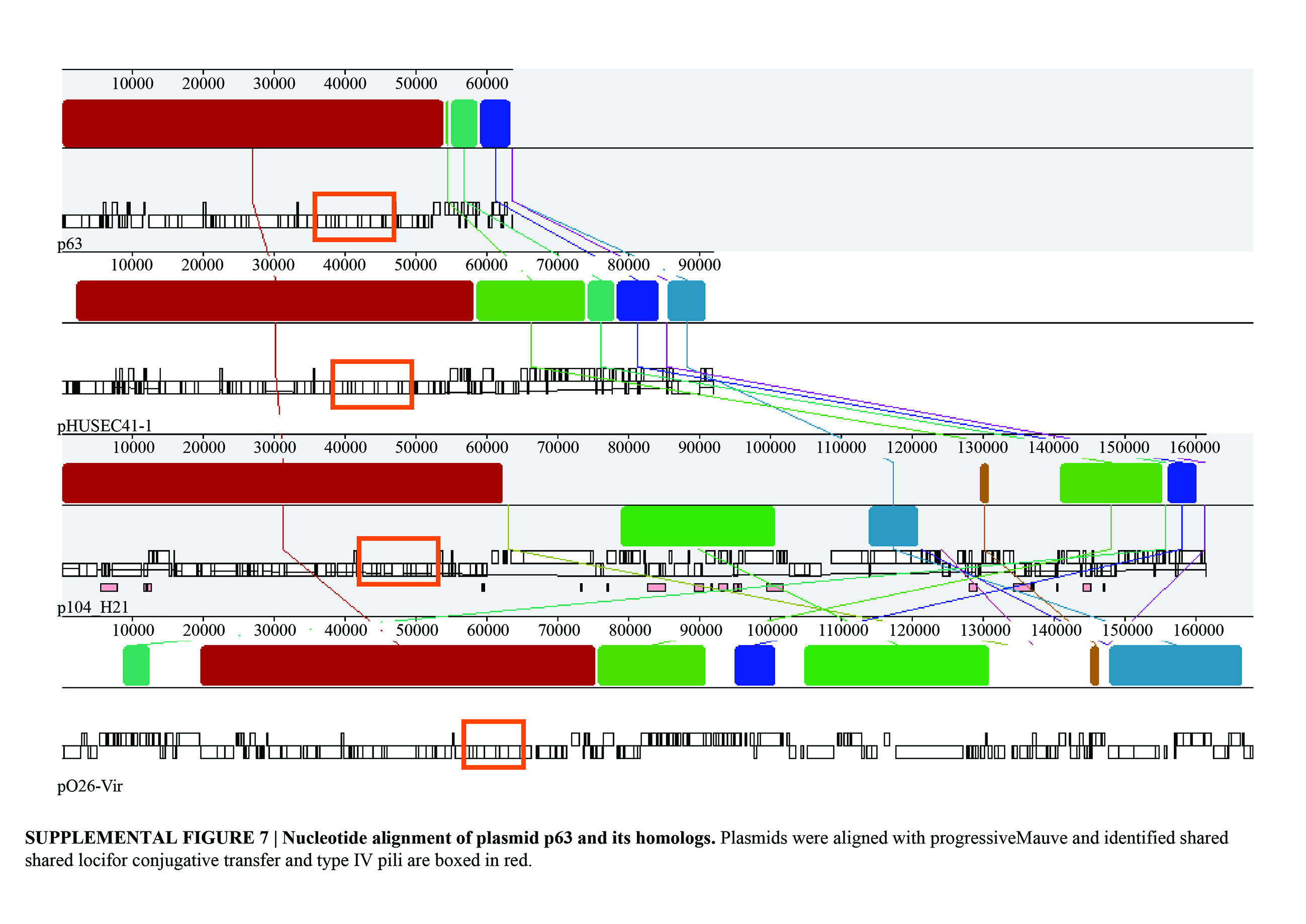

Supplement: Supplementary file 11 [file Image7.TIF]
